# Statins Modulate Microenvironmental Cues Driving Macrophage Polarization in Simulated Periodontal Inflammation

**DOI:** 10.3390/cells12151961

**Published:** 2023-07-29

**Authors:** Waleed Alkakhan, Nico Farrar, Vanessa Sikora, Pinar Emecen-Huja, Sarandeep S. Huja, Özlem Yilmaz, Subramanya N. Pandruvada

**Affiliations:** 1 Department of Oral Health Sciences, James B. Edwards College of Dental Medicine, Medical University of South Carolina, 173 Ashley Avenue, Charleston, SC 29425, USAyilmaz@musc.edu (Ö.Y.); 2 Division of Periodontics, James B. Edwards College of Dental Medicine, Medical University of South Carolina, 173 Ashley Avenue, Charleston, SC 29425, USA; emecenh@musc.edu; 3 Division of Orthodontics, James B. Edwards College of Dental Medicine, Medical University of South Carolina, 173 Ashley Avenue, Charleston, SC 29425, USA; huja@musc.edu

**Keywords:** periodontal disease, host immune response, macrophage polarization, statins, adjunctive therapy

## Abstract

Periodontal disease (PD) is a chronic inflammatory disorder characterized by the destruction of connective tissue, tooth loss, and systemic infections. Clinically, treatment of PD includes control of the etiologic factors via several modalities: initial therapy including scaling and root planing (SRP), corrective phase of surgical treatment, both with and without adjunct antimicrobial/pharmacological agents, followed by a maintenance/supportive periodontal therapy phase. Each treatment phase aims to control oral biofilm by addressing risk factors and etiology. Monotherapy of systemic antibiotics is insufficient compared to their use as an adjunct to SRP. The critical issue of systemic antimicrobial usage includes adverse patient outcomes and increased bacterial resistance. Therefore, alternative adjuncts to periodontal therapy have been sought. Statins are widely prescribed for the treatment of hypercholesterolemia and cardiovascular disease. Statins have demonstrated anti-inflammatory properties and immunomodulatory effects, and a few retrospective studies showed that statin patients exhibit fewer signs of periodontal inflammation than subjects without the medication. Despite the available clinical studies on the local administration of statins for PD, no studies have reported the macrophage polarization response. We have developed a gingival fibroblast–macrophage co-culture model to track macrophage response when exposed to a battery of microenvironmental cues mimicking macrophage polarization/depolarization observed in vivo. Using our model, we demonstrate that simvastatin suppresses macrophage inflammatory response and upregulates tissue homeostasis and M2 macrophage markers. Our findings support the usage of statins to mitigate periodontal inflammation as a valid strategy.

## 1. Introduction

*Periodontal disease* (PD) is a chronic inflammatory condition induced by bacterial aggregation and is characterized by the progressive destruction of the periodontium due to pathogenic invasion [1,2,3]. It is implicated that three periodontal pathogens, Aggregatibacter actinomycetemcomitans, *Porphyromonas gingivalis*, and *Tanneralla forsythia*, are seen as risk factors in periodontal disease. These bacterial pathogens have commonalities, including that they are all Gram-negative and produce lipopolysaccharides (LPS), can invade mucosal barriers and sequester in epithelial cells, and they all produce factors that enable them to evade the immune system [4,5,6]. The focus of these virulence factors is on how they modulate/regulate the host responses [7]. The host inflammatory response leads to the loss of connective tissue attachment, alveolar bone, and potential tooth loss [8]. The innate immune response engages in the pathogenesis of periodontitis, specifically including the role of cytokines and mediators of inflammation. The inflammatory process triggers the release of growth factors which recruit different innate immune system cells [6,9].

A harmoniously balanced oral cavity microbiome contributes to overall human health. However, a breach in this relationship causes diseases such as periodontal disease. Periodontal pathology becomes evident when bacterial dental plaque and biofilm development elicit an inflammatory response around the gingiva. The bacterial challenge remains the etiologic stimulus for susceptible hosts to develop periodontitis, and the inability of the host to resolve inflammation is critical in disease progression. Understanding the role of uncontrolled host inflammation in conjunction with the dysbiosis of microbial biofilm communities will help studies focus on treatment measures of periodontal disease. Different macrophage phenotypes are integral in chronic inflammatory conditions, including periodontal infections.

In periodontal tissues, macrophages comprise up to 30% of the cells in the cellular infiltrate of human periodontal disease. *Resident macrophages* are phagocytic and antigen-presenting cells differentiating from circulating peripheral blood monocytes [10]. They are actively involved in the regulatory functions of the innate and adaptive immune systems. Macrophages derived from different origins maintain their unique gene expression valuable in assessing polarization. In vitro, the stimuli used to differentiate cells are a granulocyte-macrophage colony-stimulating factor, GM-CSF, or platelet factor-4 (CXCL4) produced by stromal cells or within blood and tissues. They prime the macrophages to induce their differentiation, an essential further activation process into pro- or anti-inflammatory states. Studies have shown proliferation of macrophages through CSF-1 leads to a homeostatic or anti-inflammatory M2-like phenotype. GM-CSF proliferation leads to an M1-like inflammatory phenotype. Classically, activated M1 macrophages induce pro-inflammatory responses and are active during the inflammatory phase. They are highly aggressive against bacterial infiltrates and secrete further inflammatory mediators. Concerning Th1 cell-related cytokines (IFN-γ), LPS lead M1 macrophages to secrete inflammatory cytokines such as tumor necrosis factor (TNF-α), IL1β, and IL6. The result is a high capacity to present antigens and produce toxic intermediates such as nitric oxide (NO) or reactive oxygen intermediates (ROI). Alternatively, activated M2 macrophages induce anti-inflammatory responses and are active during the resolution phase of inflammation. Their functions include regulating immunity, maintaining tolerance, tissue repair, and wound healing. M2 macrophages are primed by stimulating Th2 cell-related cytokines such as IL4 and IL13, further secreting IL4 and IL10 anti-inflammatory cytokines [11]. Despite the dichotomy of M1 and M2 macrophages to Th1 and Th2 cells, there is the specific expression of chemokine components that direct the differentiation of macrophages. These can be further investigated with transcriptional profiling and proteomic analysis, adding potentially new dimensions to the plasticity of macrophages [12].

Mechanical biofilm control by physical disruption and elimination of dental biofilms (scaling and root planning or SRP) can be effectively accomplished with standard oral hygiene practices for tooth brushing and interdental cleaning. With our understanding of the fastidious early and late colonizers of periodontal biofilms, persistently poor plaque control can lead to re-colonization [13]. As a result, adjunctive therapies have been proposed to improve the clinical and microbiological outcomes of treatments. Chemical adjunctive therapies can be achieved by different mechanisms of action, including preventing bacterial adhesion, avoiding bacterial growth/co-aggregation, eliminating an already established biofilm, and altering the pathogenicity of the biofilm [14]. Systemic antimicrobials against subgingival biofilm have been shown to affect periodontal therapy when used as adjunct therapies. These therapies are ineffective due to the tolerance developed by highly resistant biofilms [15]. The critical issue related to systemic antimicrobial usage includes adverse effects for patients and increased bacterial resistance [16]. While antimicrobials target bacterial species, new research on alternative adjunct therapy focuses on host modulation. Researchers have been exploring the most prescribed medications, such as probiotics, photodynamic therapy, bisphosphonate use, and statins, and searching for their additional beneficial effects to be used as adjunctive therapeutic agents. Current therapies for periodontitis that target bacterial plaque have limited success; the focus needs to shift toward host modulatory agents that will promote the resolution of inflammation and restore tissue homeostasis, especially in susceptible patients with high risk for periodontitis. Data are insufficient to suggest that topical statin or alendronate gels as adjuncts to SRP or surgical therapy may improve periodontal clinical parameters and increase radiographic intra-bony defects [17]. Supplementation with probiotics or n-3 PUFAs and aspirin is a promising adjunctive therapy for periodontitis [18,19]. However, further longitudinal studies with larger samples are required to draw definitive conclusions about their therapeutic impact. Current preclinical evidence of pro-resolving mediators and complement inhibitors supports their high potential to revolutionize the treatment of periodontitis in the near future [20].

Statins are widely prescribed to treat hypercholesterolemia and cardiovascular disease. They are competitive inhibitors of HMG-CoA reductase, which causes compensatory cellular responses, including regulation of low-density lipoproteins. These drugs inhibit the synthesis of mevalonate, a precursor to the synthesis of cholesterol and isoprenoids [21]. Isoprenoids have shown the ability to reduce inflammation with specific action inhibiting IL-6-mediated inflammation. Thus, statins have demonstrated anti-inflammatory properties and immunomodulatory effects [22]. While statins have been historically used to treat cardiovascular disease, the anti-inflammatory nature of their pleiotropic effects is relative to other conditions that seemingly are linked to cardiovascular diseases. Chronic periodontal disease has been linked to cardiovascular and disease effects [23]. A retrospective study showed that patients taking statin medication exhibit fewer signs of periodontal inflammation than those without the medication [24]. Despite the study’s limitations, it served as an indicator for future research when searching for adjunctive agents in periodontal therapy. Given the interesting clinical findings and the uncertain results from population-based studies, further studies are required on how statins can modulate macrophage response and aid in periodontal treatment. We have previously shown that an oral anaerobic microbe, *P. gingivalis* infection, induces the formation of M1 macrophages in vitro and in vivo [25]. Interestingly, the in vitro studies we carried out to identify the effect of adaptive immune response using *opsonized-P. gingivalis* challenge show a dampening M1 response and a switch to a more M2-like phenotype. Interestingly, modulation of the transcription factor Kruppel-like factor 4 (KLF4) was consistently downregulated following the initiation of periodontal inflammation. Since KLF4 directs macrophage polarization to the M2 phenotype (alternative pathway), these observations suggest that more M1 macrophage (classical) phenotype is favored during PD, whereas blocking TLR signaling of *P. gingivalis* via opsonization leads to upregulated KLF4 expression, suggesting macrophage plasticity in the resolution of inflammation. It is unclear if M1 and M2 macrophages can undergo dynamic transitions between different functional states during a “physiological” inflammatory response to establish macrophage plasticity in periodontal disease. Through the in-depth study of the mechanisms regulating macrophage polarization and guiding the proportion of the M1/M2 phenotypes to control the inflammatory response, the therapeutic idea of achieving a good balance between immune defense and tissue homeostasis can be demonstrated.

Here, we developed a gingival fibroblast–macrophage co-culture in vitro model to mimic periodontal infections and expose them to statins. We evaluated the changes in gene expression throughout the polarization cycles to analyze if statins impact specific stages and markers associated with periodontal infections and inflammation. Intending to study the host response to periodontal disease and develop adjunctive drug therapies, we clarify statins’ role in periodontal disease’s inflammatory effects. Our research methodology was designed to specifically understand how macrophage skewing contributes to PD in the downstream development of an immune response and how to develop new therapeutics.

## 2. Materials and Methods

### 2.1. Cell Culturing and Priming of THP-1-Derived Macrophages

Human leukemic cell line THP-1 cells were used to generate macrophages. THP-1 cells were seeded at 300,000 cells/mL in antibiotic-free RPMI media + 10% FCS and maintained at 37 °C, 5% CO_2_ in a humidified tissue culture incubator. THP-1 cells were primed by exposure to 50 ng/mL phorbol 12-myristate (PMA) added to RPMI media + 10% FCS for 24 h. PMA was washed off, followed by a 72 h rest period in fresh media before exposure of polarizing cytokines. The PMA treatment protocol was adapted from Baxter et al. in 2019 [26].

### 2.2. Gingival Fibroblast and Macrophage Co-Cultures

The introduction of a co-culture with human primary gingival fibroblasts (HGF, ATCC) [27] and macrophage was utilized to assess macrophage plasticity in an environment replicating the periodontal microenvironment in vitro. Briefly, HGF was plated in trans-well inserts (6-well format) 72 h before transferring to macrophage cultures. Then, cells were cultured in macrophage media (RPMI 1640+Glutamax-I Medium supplemented with 5% heat-inactivated human AB serum) or as co-cultures with gingival fibroblasts (HGF cells) separated by trans-wells. Macrophage cultures, as described earlier, were subjected to a battery of cytokines mimicking inflammation response in vitro.

### 2.3. Cytokine Titration

**Figure 1:**
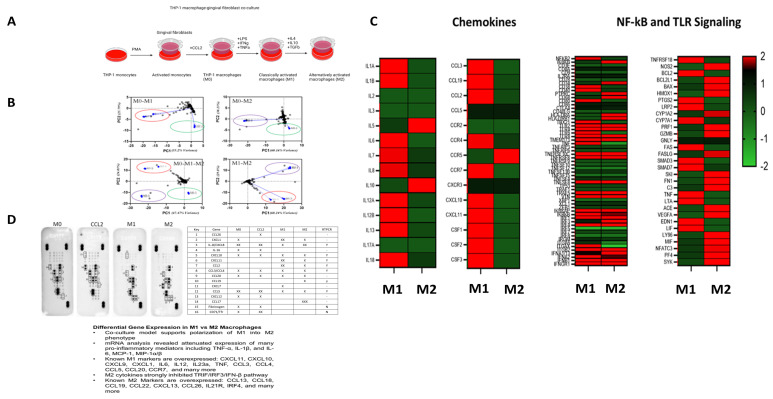
Validation of plasticity in THP-1-derived M1/M2 macrophages. (**A**) A schematic representation of treatments to differentiate THP-1 monocytes into macrophages (M0–M1–M2). (**B**) Gene expression principal component analysis clustering M0 (CCL2), M1 (LPS, IFNγ, TNFα), and M2 (IL4, IL10, TGFβ) macrophages. (n = 3) preparations per treatment group. (**C**) Heat map of averaged gene expression profiles (n = 3; data found to be significantly different were plotted as heat maps). (**D**) The proteome profiler on culture supernatants assessed chemokine secretion to validate RT-PCR analysis. Supernatants pooled from 3 cell preparations.

Following cell culturing and priming, THP-1 was sequentially exposed to microenvironmental cues at different time intervals: CCL2, LPS, IFNγ, TNFα, IL4, IL10, and TGFβ (LPS from Sigma and the rest from Peprotech) [28,29]. Untreated cells were sampled as the control at T_0_, representing monocytes before inflammatory cytokine exposure. The remaining cells were exposed to CCL2 (10 ng/mL) and rested for 2 h in the incubator. At T_1_, cells were sampled, representing monocytes activated for recruitment by CCL2. After a complete media change, cells were exposed to LPS (5 µg/mL), IFNγ (25 ng/mL), and TNFα (10 ng/mL) and incubated for 24 h, where they were sampled, representing the M1 macrophages (T_2_). Following a complete media change to clear M1 cytokines, cells were treated with IL4 (20 ng/mL), IL10 (20 ng/mL), and TGFβ (10 ng/mL), followed by incubation for an additional 24 h. Cells were sampled at T_3_, representing M2 macrophages (Figure 1 shows the schematic of cytokine treatments).

### 2.4. Simvastatin and MyD88 Inhibitor Treatments

We assessed macrophage transcripts from THP + GF alone and THP + GF + SMV. We utilized simvastatin (a synthetic derivative of a fermentation product from Aspergillus terreus) as a representative statin. Briefly, macrophage cultures were pretreated with simvastatin (MedChemExpress, 10µM, 18 h prior to polarization protocol), followed by complete media change before initiating CCL2 treatment (as shown above). Notably, simvastatin was replenished at each time point of introducing new media throughout the protocol. MyD88 homodimerization inhibitor, T6167923 [30] (MedChemExpress, 10 µM, 18 h prior to polarization protocol).

### 2.5. RNA Extraction and cDNA Synthesis

Cells were washed in PBS and lysed using the Qiagen RLT plus buffer from the lysate according to the Qiagen RNeasy plus kit protocol. Briefly, the lysate was added to a genomic DNA exclusion column and centrifuged at 10,000× *g* to remove any genomic DNA. Lysate was then diluted at a 1:1 ratio with 70% ethanol and added to a mini-spin column before another 10,000× *g* centrifugation, adhering the RNA to the column membrane and removing the buffer. RNA was washed once with RW1 buffer and twice in RPE buffer to remove impurities. RNA was then eluted using RNase-free deionized water, RNA concentrations were measured, and purity was checked using a Nanodrop-1000 spectrophotometer (ThermoFisher Scientific, Waltham, MA, USA). Samples were diluted with RNase-free water to 100 ng/mL and stored at −20 °C until cDNA synthesis.

cDNA was produced according to the Applied Biosystems high-capacity cDNA Reverse Transcription kit protocol. TaqMan gene expression reagents from a 16S ribosomal RNA and GAPDH were used as controls for the amount and quality of RNA in the cDNA synthesis. A 1.0 μg RNA template was mixed with 10× Random Primers and 25× dNTP mix (100 mM) at 25 °C for 10 min. Reactants were transferred to a thermal cycler and heated to 25 °C for 10 min so primers could anneal to the RNA template. One-unit MultiScribe Reverse Transcriptase enzyme was added, and the reactants were heated to 37 °C for 120 min. The responses were stopped by heating to 85 °C for 5 min before RT-PCR.

### 2.6. NanoString Analysis

NanoString nCounter gene expression system was used for RNA analysis for immune response. The nCounter gene expression system is a multiplexed probe detection system that relies on a probe library constructed with two sequence-specific probes for each gene of interest. Following the manufacturer’s protocols, samples (200 µg RNA per sample) were hybridized, and products were run on the nCounter Preparation Station (NanoString Technologies, Seattle, WA, USA) (n = 3 independent). A human immune response V2 panel was utilized. Transcript counts were normalized to the geometric means of spiked-in positive controls, negative controls, and built-in housekeeping genes. Differential gene expression and pathway analysis were performed using the nSolver Advanced Analysis Software version 2.0.134 (NanoString Technologies). Normalized mRNA transcript counts and pathway analysis are included in Supplementary Table S1.

### 2.7. Quantitative Real-Time PCR for mRNA

cDNA was synthesized utilizing TaqMan Random Hexamers and Reverse Transcription Reagents (Applied Biosystems, Waltham, MA, USA), per the manufacturer’s protocols, and was amplified using TaqMan primer probes and TaqMan Fast Advanced Master Mix on the StepOnePlus System (Applied Biosystems), per the manufacturer’s protocols. *GAPDH* was used as an endogenous control gene, and relative quantification of mRNA was performed by the 2^−ΔΔCT^ method. Assay was performed with two technical replicate reactions.

### 2.8. Flow Cytometry

Phenotyping of macrophages was performed in 1% BSA and 3% human serum PBS according to standard methods using a panel of antibodies targeting CD68 (R and D Systems Cat# IC20401P, Minneapolis, MN, USA, RRID: http://scicrunch.org/resolver/AB_2074835, accessed on 28 April 2023), CD163 (R and D Systems Cat# FAB1607P, RRID: http://scicrunch.org/resolver/AB_2074536, accessed on 28 April 2023), and CD206 (R and D Systems Cat# FAB25342P, RRID: http://scicrunch.org/resolver/AB_10889015, accessed on 28 April 2023); antibodies all from R and D Systems. Data were analyzed with Flowing Software (v2.0, University of Turku, Turku, Finland) or FACSDiVa software (6.0, BD Biosciences, San Jose, CA, USA) and represented, when required, with a logical display [25].

### 2.9. Statistical Analyses

NanoString technologies were used for RNA analysis for immune response genes. Candidate genes were validated using Human Immune Response TaqMan RT-PCR Array and a Proteome Profiler Chemokine Array to compare gene/protein expression at different macrophage polarization stages during inflammation. The statistical evaluation of the data collected focused on descriptive statistics for macrophages’ various phenotypic and functional markers. The comparisons were derived from *triplicate* determinations of each cell preparation before and after the challenge with chemokines. Data containing continuous variables were compared among the conditions using ANOVA and the Holm–Sidak post hoc multiple comparisons tests (Graphpad Prizm) for normally distributed data. An adjusted α level of 0.05 was considered to be significantly different.

## 3. Results

Gingival fibroblasts impact the inflammatory mediators during periodontal disease. Therefore, adding gingival fibroblast co-culture enables us to understand macrophage response more accurately through cell-to-cell interactions to mimic periodontal disease in-vivo, demonstrating the polarization kinetics of M1 and M2 macrophages (Figure 1A). NanoString nCounter analysis using an immune response panel has further established the successful transformation of macrophage polarization phenotypes at the tested time points. The immune response panel with pre-hybridized primers for 570 genes showed how distinct each phenotype is after exposure to relevant cytokine cues, as evidenced by principal component analysis (Figure 1A). RT-PCR analysis for macrophage genes benchmark validation (Supplementary Figure S1) for the M1 macrophages demonstrated expression of CASP4, GAS6, and HEY2 in ten-, five-, and forty-fold differences, respectively, compared to M0 macrophages. M2 macrophages showed expression of KLF4, CD9, and CD163 in five-, two-, and six-fold differences, respectively, when compared to M0. These validate the in vitro model of macrophage polarization. These results suggest that our model has successfully recapitulated the macrophage polarization from M0 to M1 to M2 states. NF-kB and TLR signaling pathways exhibit differentially expressed genes and macrophage benchmarks in the gingival fibroblast co-culture models (Figure 1C). Up-regulated genes in M1 polarization include CCR7, CD40, CXCL1, CXCL11, IL6, CXCL10, CXCL9, and many more. Similarly, we detected overexpression of common M2 markers such as CCL13, CCL18, CCL19, CCL22, CXCL13, and others in our in vitro model. Similar to pathogen- and host-related mediator activation of the NF-kB and TLR signaling pathways, our data are concurrent with how these pathways affect macrophages. Crosstalk between NF-kB and TLR signaling aids in the ability of macrophages to demonstrate their pro-inflammatory or anti-inflammatory states. Specifically, MyD88 and TRIF utilization demonstrated the link between the NF-kB pathway’s requirement for TLR signaling. As evidenced by the expression profile, our data demonstrated that the important element of macrophage polarization is the ability to mount an effective immune response while protecting the host from the undesired effects of hyperinflammatory states (Figure 1C). To further assess the initial findings of phenotypic change between macrophage polarized states, we conducted validation of chemokine expression using specific genes typically seen in M0, M1, and M2 macrophages (Figure 1D). The protein array demonstrates the increase and decrease of chemokines seen when polarized macrophages are in the M1 or M2 states. Here, we show an increase in CXCL1, CXCL11, and CCL2 at M1 macrophages compared to M0 or M2 macrophages. These findings support our existing knowledge of the role of M1 macrophages in a pro-inflammatory capacity. We can also see increased CCL19 and CCL17 in M2 macrophages compared to M0 to M1 macrophages, as typically seen in anti-inflammatory states. These genes upregulated in M1 and M2 macrophages represent pro-inflammatory and anti-inflammatory genes known as markers. In addition, we saw a downregulation of chemokines, such as IL-8, in the M1 state when compared to M0 and M2. These results validate a few secreted products among protein array, NanoString, and RT-PCR analyses.

**Figure 2:**
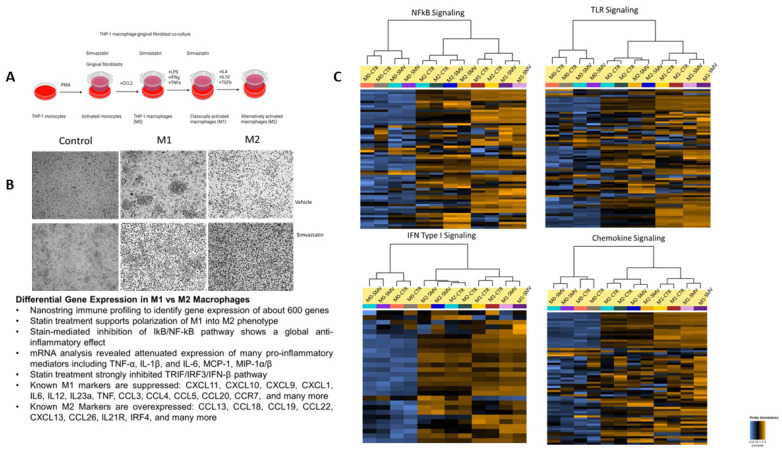
Simvastatin is a modulator of macrophage plasticity. (**A**) A schematic representation of simvastatin treatment and THP-1-macrophages (M0–M1–M2). (**B**) Morphological characteristics observed. (**C**) Unsupervised clustering and heat maps summarizing chemokine, TLR, NFkB, IFN-I signaling from M0 (CCL2), M1 (LPS, IFNγ, TNFα), and M2 (IL4, IL10, TGFβ) macrophages and were plotted as heat maps (n = 3 preparations per treatment group). See Supplementary Figure S2 for more information.

Macrophages were pretreated with simvastatin 18 h before the initiation of polarization through our model (Figure 2A). Again, simvastatin was replenished during media change, as indicated in the schematic. In the vehicle treatment group, M1 macrophages show a more stellate or spindle-like morphology, while M2 macrophages are more circular in shape. The concentration of cytokines administered alters the phenotypic morphology of the macrophages (Figure 2B), and previous studies have shown that higher concentrations of cytokines administered to M2 macrophages demonstrate stellate morphology; therefore, lower concentrations were utilized to ensure polarization to the M2 state [26]. Further, phenotype modification supports the changing morphology from M1 to M2. As depicted in Figure 2B, the M1 macrophages exhibit a spindle shape or stellate-like morphology. Compared to the vehicle treatment group, the simvastatin-treated group showed no change in the morphology of the macrophages displaying polarization in each state. Simvastatin treatment also supported successful polarization of M1 into M2 macrophage phenotype through modulation of key chemokines, TLR, NFkB, and IFN-I signaling (Figure 2C; Supplementary Figure S2). Statin-mediated inhibition of these pathways demonstrates a global anti-inflammatory effect by suppressing many pro-inflammatory mediators such as TNF-α, IL-1β, IL-6, MCP-1, and MIP-1α/β, suggesting a crosstalk between these pathways with further downregulation of M1 genes. Notably, our NanoString data highlighted the differential gene expression between M0, M1, and M2 macrophages when treated with simvastatin with a *p*-value of *p* < 0.05 (Table 1). The overall results demonstrate macrophage plasticity can be responsive to the microenvironment cues because of statin treatment.

**Figure 3:**
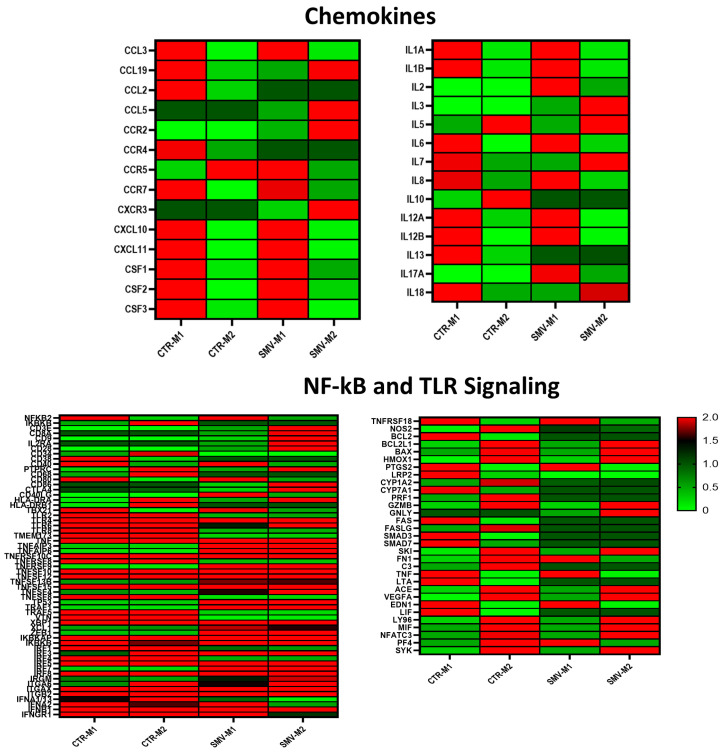
Simvastatin promotes M1 to M2 switch. RT-PCR validation of NanoString data summarizing chemokine, TLR, NFkB, signaling from M0 (CCL2), M1 (LPS, IFNγ, TNFα), and M2 (IL4, IL10, TGFβ) macrophages presented as heat maps. (n = 3) preparations per treatment group, and data found to be significantly different were plotted as heat maps.

We validated top differentially expressed genes that have demonstrated a two-fold change in expression between M1 and M2 macrophages by RT-PCR. Classical M1 markers to be suppressed include CXCL11, CXCL10, CXCL9, CXCL1, IL6, IL12, IL23a, TNF, CCL3, CCL4, CCL5, CCL20, CCR7, and others. Similarly, M2 markers, such as CCL13, CCL18, CCL19, CCL22, CXCL13, CCL26, IL21R, IRF4, and others, are overexpressed (Figure 3). Amongst those is CCL18, which had a statistically significant (*p* = 0.001) increase in expression by a fifteen-fold change during the M2 state, compared to its expression at the M1 state being the two-fold change. CCL13 is another marker that shows a statistically significant increased expression of seven (*p* = 0.024) at M2, while at M1, it only demonstrated a three-fold change. CD209 is an additional marker demonstrating the same increase in expression at M2 compared to M1. CD209 increased three-fold (*p* = 0.011) compared to its state at M1 being two-fold. These three markers, in addition to CD81, CCL19, and MALT1, represent just a few of the sixteen identified genes that have demonstrated an increase in expression during the anti-inflammatory M2 state. Exposure to simvastatin increased the expression of these genes during the anti-inflammatory state. IL6 is a prominent pro-inflammatory marker known as a hallmark of inflammation. Our data show that IL6 has a 4.71 decrease in expression in the M1 macrophages when exposed to simvastatin (*p* = 0.005). CCL20 is suppressed during the pro-inflammatory M1 state, with a significant 10-fold decrease in expression (*p* = 0.009). Amongst the suppressed genes is CCL3, demonstrating a significant five-fold decrease in expression (*p* = 0.016). Several pro-inflammatory markers in NFkB and TLR signaling pathways are decreased in the statin treatment group. These markers can aid in our ability to better understand how macrophages respond to their microenvironment, specifically in the presence of statins with anti-inflammatory properties. Further, a closer look at the markers in our data highlights potential new candidates altered in the statin treatment panel. Notably, LTA and LIF show the same expression pattern; both overexpressed in the M1 control but downregulated in the simvastatin treated M1 states; this suggests suppression of pro-inflammatory changes within our model. Comparing LTA and LIF expression during M2 states, we observed an upregulation in simvastatin-treated M2 macrophages. These unique markers demonstrate a change of expression towards the overall anti-inflammatory effect on both M1 and M2 macrophages.

**Figure 4:**
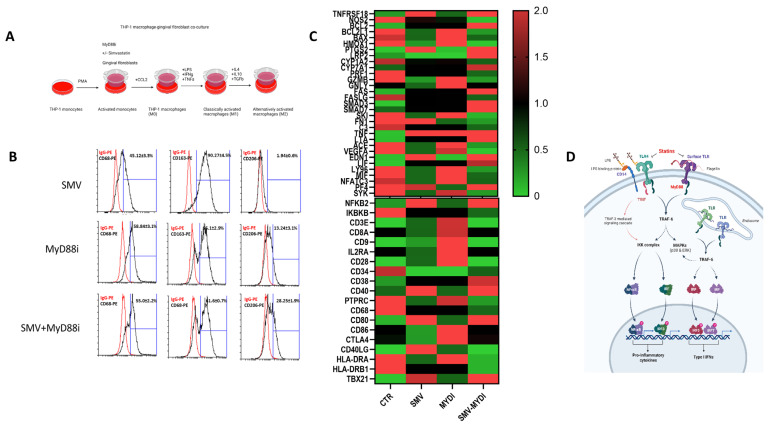
MyD88−dependent modulation of M2 polarization by simvastatin. (**A**) A schematic representation of simvastatin, MyD88 inhibitor treatment, and THP-1-macrophages (M0–M1–M2). (**B**) Cell surface antigen analysis of M2 macrophage markers CD68, CD163, and CD206 was conducted using flow cytometry. Expression levels were normalized using IgG isotype control, and values were expressed in percentage of positive cells (mean ± SD) from three independent preparations. (**C**) RT-PCR analysis and heat maps summarizing TLR and NFkB signaling from M0 (CCL2), M1 (LPS, IFNγ, TNFα), and M2 (IL4, IL10, TGFβ) macrophages. (n = 3) preparations per treatment group, and data found to be significantly different were plotted as heat maps. (**D**) Graphical summary of statin-mediated suppression of TLR and NFkB signaling.

Ligand-specific activation of TLRs recruits the adapter molecules MyD88 or Toll/IL1R (TIR) domain-containing adapter producing interferon-β (TRIF). Recruitment of MyD88 or TRIF elicits pathway-specific downstream signaling and gene transcription events, with MyD88 signaling culminating the production of pro-inflammatory mediators and TRIF signaling resulting in the production of Type I interferons. TLR4 uniquely signals through both the MyD88- and TRIF-dependent signaling cascades. We determined the relative contributions of MyD88-dependent signaling pathways and the role of simvastatin in driving TLR-mediated macrophage polarization. Using a combination of statin and MyD88 inhibitor in vitro, we show that macrophage polarization is amplified in a MyD88-dependent manner, and simvastatin treatment further augmented M2 state or tissue homeostasis (Figure 4). When macrophages were treated with either simvastatin or MyD88 inhibitor, T6167923 during the PMA withdrawal and resting phase (for 48 h) and phenotypes for macrophage M2 surface markers were assessed; an augmented CD68^hi^CD163^hi^CD206^int^ signature was evident with the statin–MyD88 inhibitor-treated cells (Figure 4B). Furthermore, macrophage-related gene expression was also confirmed by RT-PCR with marked upregulation of M2 genes in the statin–MyD88 inhibitor-treated cells (Figure 4C). These findings demonstrate a crucial mechanism of immune response modulation in PD by providing evidence in vitro that statins’ anti-inflammatory effects are MyD88 dependent (Figure 4D).

## 4. Discussion

The oral cavity is a highly complex environment of microorganisms forming biofilms. The host tissues maintain a barrier function to those biofilms by releasing mediators and signals of the immune system. Co-culture techniques allow various cell types to cultivate together, examine cell-cell interactions, and measure the levels of cytokines present [31]. In vitro models have utilized a co-culture model to answer questions regarding host immune response. Several theories have been put forth to explain the concept of macrophage plasticity [32]: (1) The concept is that different subsets of macrophages adopt different functional phenotypes, such as Ly6C+ monocytes becoming anti-inflammatory M1 macrophages and Ly6C− monocytes/tissue-resident monocytes becoming M2 macrophages. Meanwhile, in this model, resident macrophages have cytoprotective and anti-inflammatory functions, while circulating macrophages perform mainly proinflammatory functions; (2) Due to different phases of inflammation, waves of monocyte recruitment occur, dependent on other cytokine signals that are elicited at various time points, and polarization occurs when the monocytes encounter specific signals; (3) Polarized macrophages can switch from one phenotype to the other based on different conditions. The ability to demonstrate the plasticity of macrophages from one phenotype to another based on microenvironmental cues makes way for targeting specific genes during drug therapy of inflammatory diseases. Studies have shown the possibility that macrophage populations are replenished dynamically in the inflamed sites supporting the notion that M1 and M2 macrophages can exist interchangeably. We provide evidence that M1 and M2 macrophages arise from the same cells that can shift from one functional phenotype to another that serve different functions based on microenvironmental cues.

Gingival fibroblasts are a primary cell type in gingival tissues that secrete a variety of inflammatory mediators and cytokines. They modulate the innate inflammatory response of macrophages, and the literature supports negatively impacting the phagocytic response of bacteria during periodontal disease [33]. Studies have indicated the ability to interrogate bacterial interactions such as *P. gingivalis* with gingival fibroblasts in the co-culture setting. Tissue damage in periodontal disease is driven by bacteria directly, in addition to the host’s response to dysbiotic microbiota [31]. We put forth a model incorporating gingival fibroblast co-cultures to study macrophage polarization. However, our design is focused on pathogenic components rather than pathogens for a more straightforward approach. Furthermore, our group has previously shown that *P. gingivalis* can trigger this pathway in macrophages and synergize with host factors, i.e., IFNγ and extrinsic LPS, to induce significant elevations in M1-produced inflammatory mediators [25,34,35]. Furthermore, *P. gingivalis* selectively tolerates macrophage subsets that could facilitate immunopathology and marginalize immunity [21]. However, *P. gingivalis*-LPS alone can weakly activate macrophage polarization while inducing proinflammatory mediators via TLR2 engagement [36]. Commercial *P. gingivalis*-LPS potency differs from batch and method of purification, with inconsistent results among research groups, and has a significant difference in the relative potencies between *Escherichia coli* LPS and *P. gingivalis*-LPS in inducing proinflammatory cytokines, where *P. gingivalis*-LPS is less effective [21]. We have employed *E. coli* LPS in our experiments. We will extend our study by incorporating oral pathogen-infected gingival fibroblasts in our model to mimic periodontal disease accurately.

Here, we report the upregulation of classic proinflammatory genes and chemokines, such as IL6, during M1 polarization. This is supported by many studies indicating an increase in IL1β and IL6 secreted by M1 macrophages to induce the expression of MMPs in human gingival fibroblasts to destroy periodontal tissues [37]. A synergistic role is incorporated between gingival fibroblasts and M1 macrophages as IL6 is upregulated in this state. The premise of introducing statins to our in vitro model is based on the pleiotropic effects that showed decreased C-reactive protein levels in the plasma of patients taking the drug [38,39]. These opened the door for further investigation of these anti-inflammatory effects and the eventual discovery of statin’s impact on mevalonate pathway inhibition. More specifically, Sheridan et al. have summarized the immunomodulatory effects of statins on macrophages [40]. The group reiterated the effects of different statins, including simvastatin, to have anti-inflammatory effects through various mechanisms. Simvastatin effects on macrophages include suppression of tissue factor activity accompanied by a diminution in tissue factor mRNA expression, inhibition of IFN-γ-induced upregulated mRNA expression of chemokines such as MCP-1, MIP-1a, MIP1b, CCR1, CCR2, and CCR5, and a reduction of MCP-1 protein expression [40].

Our data from simvastatin treatment show that statins’ anti-inflammatory effects are mediated through TLR, NFkB, and IFN Type 1 signaling pathways (Figure 2 and Figure 3). The suppression of proinflammatory cytokines coincides with how these pathways communicate: NFkB signaling influences TLR and IFN signaling due to the pattern recognition receptors’ (PPRs) involvement in these pathways. Stimulation of TLR4 with LPS through MyD88 leads to activation of NFkB, MAPK, and IRF5 pathways, while TLR signaling will also lead to the production of Type 1 IFNs. Signal crosstalk after cell stimulation is evident, and the ability of macrophages to be impacted by statins through these mechanisms is supported by our data. The anti-inflammatory effects of statins are through these pathways due to the downregulation of M1 genes and upregulation in the M2 form. Statin-mediated inhibition of the mevalonate pathway and, thus, isoprenoids result in the attenuation and degradation of NFkB inhibitor protein IkB; however, the exact link is not fully understood. Inhibition of these pathways, validated using mRNA and protein analyses, reveals the suppression of proinflammatory cytokines, including TNFα, IL1β, and IL6. The crosstalk between different pathways includes the role of MyD88 signaling that, when inhibited during the polarization program, further augmented statins’ inhibitory effects, primarily mediated through TLR signaling (Figure 4). MyD88 and TRIF are nonredundant signaling pathways in inflammation, but MyD88 is the essential adaptor molecule for the transduction of early TLR4-induced inflammatory signaling. Significant gene induction of all inflammatory mediators depended on intracellular signal transduction by MyD88. LPS induction of MyD88 was TLR4-dependent on the hematopoietic and nonhematopoietic cells. Conversely, no induction of TRIF mRNA was detectable. The reduction of IFN-B expression resulted in decreased STAT1 and reduced IL6. Sheridan et al. discuss these pathways in detail, supporting the ability for statin-mediated anti-inflammatory effects in many ways [40]. Interestingly, our data indicate that several genes are less significantly known in M1 states, such as PF4 and FN1. PF4 and FN1 are expressed during phases of wound healing. M2 upregulated genes include CCL19, CD19, CD28, and HLA-DRA, which display red in the M2 panels and green in the M1 panel (Figure 3). The differential gene expression pattern between the two polarized states indicate that gingival fibroblast co-culture systems support polarization in our model. Simvastatin has been shown to upregulate CD9 expression, leading to reduced TNF-α and MMP-9 production. CD9 is a known anti-inflammatory marker. Studies from knockout mice models show that CD9 prevents the formation of CD14/TLR4 complexes, and the mechanism could be due to inhibitory action on protein prenylation [41]. CD9 is upregulated when M2 macrophages are exposed to simvastatin compared to no simvastatin treatment.

Statin-mediated anti-inflammatory modulation through IFN-γ signaling pathways has shown immunosuppressive impacts through decreased expression of class II transactivator (CIITA) [42]. Studies regarding this mechanism have indicated insights into yet another pathway for macrophages to be impacted by statins. The exact mechanisms need further investigation, but findings, including the downregulation of STAT1 and IRF1 transcription factors, are promising in understanding statin mechanisms. Our kinetic model modulated these factors in the statin treatments. With the pathways described above, we show that statins impact macrophage differentiation, and our kinetic model demonstrates the downregulation of overall proinflammatory effects and upregulation of anti-inflammatory effects.

Furthermore, our inflammatory model supports our hypothesis that the same macrophage transitions from M0 to M1 and M2 when exposed to microenvironmental cues replicating a periodontal inflammation. Interestingly, we found that phenotype switching is totally dependent on CCL2 pretreatment. It is interesting to note that inflammatory macrophages depend on two main chemokines involved in the inflammation-dependent recruitment of Ly6C+ and Ly6C-monocyte subsets CCL2/CCRS and CX3CL1/CX3CR1. During inflammation, mesenchymal cells can produce CCL2, triggering the release of monocytes from the tissue into the bloodstream. Mouse models have demonstrated that CCR2 is required for Ly6C+ monocytes, which are important during bacterial infection. The absence of CX3CR1 reduces Ly6C-monocyte numbers, which are linked to the differentiation of M2 macrophages, and our in vitro model further confirms the participation of CCR5 in phenotype switching [32].

The introduction of statin treatment in a gingival fibroblast co-culture environment showed suppression of pathways that lead to the pleiotropic effects of statins. Our novel experimental design allowed us to identify genes potentially driving macrophage plasticity. Suppression of TLR4, NF-kB, and IFN pathways and modulating expression of proinflammatory cytokines give way to further exploring the anti-inflammatory effects of statins in periodontal disease. We have identified numerous gene targets affected by statins that can be further evaluated to assess how statins modulate proinflammatory signaling in vivo, such as in a ligature-induced periodontitis mouse model. Systemic administration followed by local administration of statins in vivo and analyzing gene expression when subjected to periodontal disease conditions can give us insight into future utilization of drug therapy. Although our experiments support statin-mediated anti-inflammatory effects, there are paradoxical findings of studies demonstrating increased secretion of proinflammatory cytokines with statins [40]. However, different experimental designs and the complex nature of cell-to-cell interactions in vitro make standardizing these effects difficult; the heterogeneity of experimental designs, cell types, drug types, doses, duration times, and treatment times all contribute to variations in investigating the statin effect on macrophage responses. Micro-environmental cues and cell culture environments can modify macrophage polarization and expression. With differences in experimental designs, standardizing these effects can be contradicting. Statins can be pursued as a treatment strategy in periodontal disease in vivo to validate our findings. Again, future clinical research can help answer the questions regarding statin usage to boost immune response in periodontal disease. Studies are ongoing to determine which of and to what extent these pleiotropic effects account for the clinical benefits of statin therapy beyond cholesterol lowering. These effects may vary significantly from patient to patient, depending on underlying systemic conditions and comorbidities. Therefore, cell-specific drug targeting therapies are required to further elaborate on statin-mediated effects of macrophages in the presence of periodontal disease.

## 5. Conclusions

Periodontal disease is characterized by the destruction of connective tissue, tooth loss, and systemic infections. Statins have demonstrated anti-inflammatory properties and immunomodulatory effects, and a few retrospective studies showed that statin patients exhibit fewer signs of periodontal inflammation than subjects without the medication. Despite the available clinical studies on the local administration of statins for PD, no studies have reported the macrophage polarization response. We have developed a gingival fibroblast–macrophage co-culture model to track macrophage response when exposed to a battery of microenvironmental cues mimicking macrophage polarization/depolarization observed in vivo. Our model demonstrates that simvastatin suppresses macrophage inflammatory response and upregulates tissue homeostasis and M2 macrophage markers. Our research is designed to understand how macrophage skewing contributes to periodontal disease and, more specifically, to the downstream development of an immune response to PD-associated bacterial components and the development of new therapeutics. With a clear understanding of proinflammatory and anti-inflammatory mechanisms related to statin therapy, other inflammatory conditions can be better implied. Together, our work supports the usage of statins to mitigate periodontal inflammation as a valid strategy.

## Figures and Tables

**Table 1:** Top differentially regulated genes in simvastatin-treated macrophages (M0, M1, and M2 populations).

Probe Name	SMV-M0 vs. PBS	*p* Value	Probe Name	SMV-M1 vs. PBS	*p* Value	Probe Name	SMV-M2 vs. PBS	*p* Value
S100A8	38.06	0.027686	S100A8	10.59	0.036081	CCL18	15.45	0.001295
FCGR2A	10.63	0.029277	CCL22	10.39	0.004261	CCL13	6.7	0.024009
S100A9	8.39	0.008924	FCGR2A/C	6.78	0.0072	CD163	4.8	0.01434
C1QA	7.4	0.040231	LILRA5	6.17	0.005036	PLAU	4.68	0.003134
GPR183	5.67	0.004147	LILRB2	5.92	0.006537	C1QA	3.63	0.025428
HLA-DMA	5.61	0.024556	CD4	5.82	0.006156	CCL8	3.53	0.022808
C2	4.56	0.024926	PLAU	5.29	0.0349	FCGR2A	3.48	0.01716
PRDM1	4.48	0.007849	IL12B	4.28	0.013388	CD209	3.26	0.011215
CXCL13	4.18	0.011733	IL7R	4.14	0.038113	CCL7	3.22	0.033946
CXCR4	4.12	0.034036	PDCD1LG2	4.14	0.003916	C1QB	2.96	0.005794
CD4	4.11	0.005522	TNFRSF9	3.83	0.019835	CMKLR1	2.8	0.041894
IL10RA	3.81	0.013677	STAT4	3.73	0.002715	CLEC7A	2.77	0.026241
CSF3R	3.52	0.014897	CSF3R	3.6	0.014988	FN1	2.71	0.014239
CCR2	3.43	0.010673	TGFBI	3.57	0.014425	LILRB4	2.7	0.042805
TLR7	3.38	0.001575	MALT1	3.28	0.003837	PECAM1	2.66	0.050232
CD74	3.31	0.017415	CMKLR1	3.25	0.017735	EDNRB	2.57	0.042912
MRC1	3.13	0.034188	LILRA6	3.16	0.006047	CD14	2.5	0.02285
PLAU	3.08	0.02921	CXCR4	3.1	0.030638	MBP	2.21	0.0013
CYBB	3.07	0.03047	PRDM1	3.05	0.010191	CYBB	2.2	0.016844
HLA-DRB1	2.95	0.007501	CCL13	3.01	0.005962	IL8	2.14	0.004803
AHR	2.93	0.031967	CD45R0	3.01	0.008954	CD4	2.11	0.023022
IL13RA1	2.64	0.018498	CCR7	2.95	0.002202	TAGAP	2.09	0.021705
LY96	2.64	0.007948	CEBPB	2.79	0.019578	CXCL11	−2.04	0.043097
HLA-DRB3	2.63	0.023405	CYBB	2.71	0.020105	BATF3	−2.16	0.014366
ICOSLG	2.51	0.009908	CTLA4_all	2.66	0.048885	CD83	−2.24	0.001721
BST1	2.47	0.003166	PTPRC_all	2.62	0.046491	LAMP3	−2.27	0.026008
IL6R	2.4	0.018439	CFP	2.44	0.04957	IL2RG	−2.6	0.012534
CMKLR1	2.31	0.048872	GBP1	2.4	0.00151	LEF1	−2.6	0.024443
CTSS	2.27	0.021659	TLR8	2.3	0.048504	CCL19	−3.04	0.022862
CASP1	2.25	0.034348	CCL18	2.29	0.035811	CXCL9	−3.54	0.032645
SYK	2.22	0.020045	CD7	2.26	0.030503			
IL10	2.07	0.041441	CD209	2.12	0.048095			
CCR1	2.05	0.014757	LAMP3	2.07	0.004902			
CD3EAP	−2.02	0.027717	TGFBR1	2.04	0.032097			
IL2RB	−2.05	0.021742	KCNJ2	2.01	0.021187			
ITGAX	−2.08	0.002873	NLRP3	−2.02	0.040695			
SLC2A1	−2.09	0.014473	CASP3	−2.06	0.015791			
PPARG	−2.16	0.018561	TNFSF4	−2.15	0.022204			
HAVCR2	−2.21	0.039523	CCND3	−2.21	0.015653			
TRAF1	−2.23	0.023926	HLA-DPB1	−2.32	0.014342			
CD276	−2.25	0.036625	LEF1	−2.35	0.043237			
TNFSF4	−2.27	0.009855	ICAM3	−2.39	0.011741			
GNLY	−2.52	0.01472	IL1B	−2.45	0.016473			
CD3D	−2.53	0.0419	LTA	−2.49	0.004071			
ITGA5	−2.58	0.043861	IL1RN	−2.55	0.006762			
TGFB1	−2.65	0.007877	CCRL2	−2.68	0.007603			
MSR1	−2.8	0.011925	CD22	−3.79	0.003046			
MAP4K4	−3.02	0.000654	SPP1	−3.95	0.006159			
BATF	−3.12	0.004713	CCL4	−4.43	0.00182			
CXCL11	−3.21	0.0111	IL6	−4.71	0.005253			
NT5E	−3.22	0.029303	CCL3	−5.23	0.01609			
EGR2	−3.67	0.012479	TMEM173	−5.47	0.023874			
CCL5	−3.79	0.041973	CCL20	−10.65	0.009047			
IL1RN	−3.99	0.043022						
PLAUR	−4.29	0.004858						
CD22	−4.73	0.007914						
CSF1	−5.17	0.039117						
CCL20	−5.57	0.023471						
IL1B	−13.73	0.040456						

## Data Availability

The main data supporting the results in this study are available within the manuscript and its Supplemental Information. The raw and analyzed datasets generated during the study are available from the corresponding author on reasonable request.

## Supplementary Materials

The following are available online at https://www.mdpi.com/article/10.3390/cells12151961/s1; Figure S1: RT-PCR analysis for macrophage benchmark genes; Figure S2: Unsupervised clustering and heat maps summarizing chemokine, TLR, NFkB, IFN-I signaling from M0, M1, and M2 macrophages; Table S1: Annotated genes on NanoString nCounter immunology panel.
