# Supplementary material for: Statins Modulate Microenvironmental Cues Driving Macrophage Polarization in Simulated Periodontal Inflammation
Source: Cells. 2023 Jul 29;12(15):1961. doi: 10.3390/cells12151961 (PMC10417531; doi:10.3390/cells12151961)
Supplement: Supplementary file 1 [file cells-12-01961-s001.zip › Figure S1.pdf]

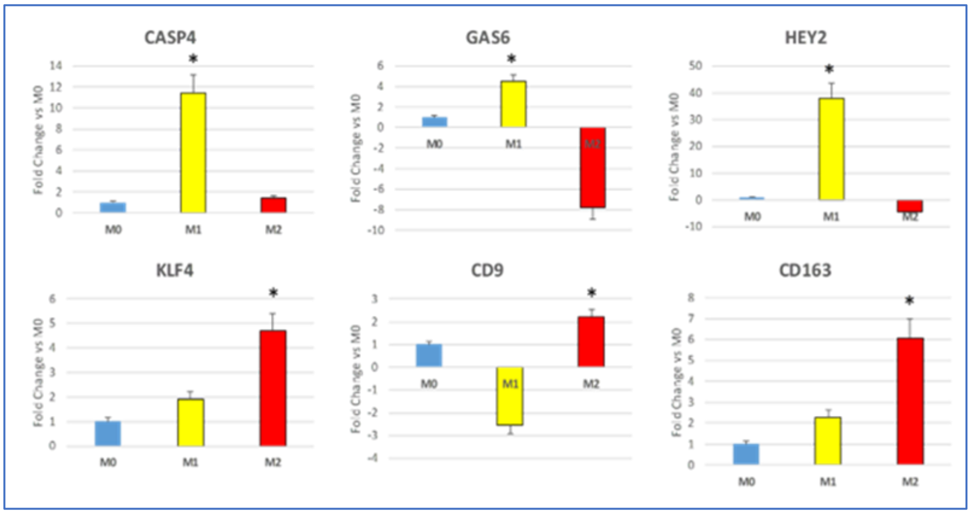

Figure S1. RT-PCR analysis of benchmark genes. Validation of M0-M1-M2 profile switching through macrophage benchmark genes from M0 (CCL2), M1 (LPS, IFN$_{\gamma}$, TNFα), and M2 (IL4, IL10, TGFβ) macrophages. (n=3 preparations per treatment group).
