# Supplementary material for: Statins Modulate Microenvironmental Cues Driving Macrophage Polarization in Simulated Periodontal Inflammation
Source: Cells. 2023 Jul 29;12(15):1961. doi: 10.3390/cells12151961 (PMC10417531; doi:10.3390/cells12151961)
Supplement: Supplementary file 1 [file cells-12-01961-s001.zip › Supplementary Table S1.pdf]

Supplementary Table S1. Summary of Annotations

Annotation	# Genes
Adaptive Immune System	141
Apoptosis	54
Autophagy	11
B cell Receptor Signaling	35
Cell Adhesion	60
Chemokine Signaling	63
Complement System	39
Cytokine Signaling	259
Hemostasis	73
Host-pathogen Interaction	252
Immunometabolism	32
Inflammasomes	8
Innate Immune System	201
Lymphocyte Activation	245
Lymphocyte Trafficking	21
MHC Class I Antigen Presentation	39
MHC Class II Antigen Presentation	14
NF-κB Signaling	62
NLR signaling	64
Oxidative Stress	36
Phagocytosis and Degradation	48
T Cell Receptor Signaling	61
TGF-β Signaling	9
Th1 Differentiation	14
Th17 Differentiation	31
Th2 Differentiation	17
TNF Family Signaling	49
TLR Signaling	73
Transcriptional Regulation	53
Treg Differentiation	10
Type I Interferon Signaling	28
Type II Interferon Signaling	36

Official Symbol	Accession	Alias or Previous Symbol
**ABCB1**	NM_000927.3	PGY1, MDR1, CLCS, P-gp, CD243, GP170, ABC20
**ABL1**	NM_005157.3	ABL, JTK7, c-ABL, p150
**ADA**	NM_000022.2	
**AHR**	NM_001621.3	bHLHe76
**AICDA**	NM_020661.1	HIGM2, CDA2, ARP2, AID
**AIRE**	NM_000383.2	APECED, PGA1, APS1
**APP**	NM_000484.3	AD1
**ARG1**	NM_000045.2	
**ARG2**	NM_001172.3	
**ARHGDIB**	NM_001175.4	RAP1GN1, GDIA2, GDID4, Ly-GDI, RhoGDI2
**ATG10**	NM_001131028.1	APG10L, DKFZP586I0418, FLJ13954
**ATG12**	NM_004707.2	APG12L, APG12
**ATG16L1**	NM_198890.2	APG16L, ATG16L, WDR30, FLJ10035, ATG16A
**ATG5**	NM_004849.2	APG5L, ASP, APG5, hAPG5
**ATG7**	NM_001136031.2	APG7L, GSA7, DKFZp434N0735
**ATM**	NM_000051.3	ATA, ATDC, ATC, ATD, TEL1, TELO1
**B2M**	NM_004048.2	
**B3GAT1**	NM_018644.3	CD57, LEU7, GlcAT-P, HNK-1, NK-1
**BATF**	NM_006399.3	B-ATF, SFA-2, BATF1
**BATF3**	NM_018664.2	JUNDM1, SNFT, JDP1
**BAX**	NM_138761.3	BCL2L4
**BCAP31**	NM_005745.7	DXS1357E, BAP31, 6C6-Ag, CDM
**BCL10**	NM_003921.2	CARMEN, CIPER, mE10, c-E10, CLAP
**BCL2**	NM_000657.2	Bcl-2
**BCL2L11**	NM_138621.4	BOD, BimL, BimEL, BIM
**BCL3**	NM_005178.2	D19S37, BCL4
**BCL6**	NM_001706.2	ZNF51, ZBTB27, LAZ3, BCL5, BCL6A
**BID**	NM_001196.2	
**BLNK**	NM_013314.2	SLP65, Ly57, SLP-65, BLNK-s
**BST1**	NM_004334.2	CD157
**BST2**	NM_004335.2	CD317, tetherin
**BTK**	NM_000061.1	AGMX1, IMD1, ATK, XLA, PSCTK1
**BTLA**	NM_181780.2	BTLA1, CD272
**C14orf166**	NM_016039.2	CGI-99, RLLM1, RTRAF
**C1QA**	NM_015991.2	
**C1QB**	NM_000491.3	
**C1QBP**	NM_001212.3	HABP1, gC1Q-R, gC1qR, p32, SF2p32
**C1R**	NM_001733.4	
**C1S**	NM_001734.2	
**C2**	NM_000063.3	

**C3**	NM_000064.2	CPAMD1
**C4A/B**	NM_007293.2	CPAMD2, C4S, CO4, C4, C4A3, C4A2, C4A4, C4A6, C4B, RG/CPAMD3, C4F, CO4, C4B1, C4B3, CH
**C4BPA**	NM_000715.3	C4BP
**C5**	NM_001735.2	CPAMD4
**C6**	NM_000065.2	
**C7**	NM_000587.2	
**C8A**	NM_000562.2	
**C8B**	NM_000066.2	
**C8G**	NM_000606.2	
**C9**	NM_001737.3	
**CAMP**	NM_004345.3	CAP18, FALL39, FALL-39, LL37
**CARD9**	NM_052813.4	
**CASP1**	NM_001223.3	IL1BC, ICE
**CASP10**	NM_032977.3	MCH4
**CASP2**	NM_032982.2	NEDD2, ICH1
**CASP3**	NM_032991.2	CPP32, CPP32B, Yama, apopain
**CASP8**	NM_001228.4	MCH5, MACH, FLICE, Casp-8
**CCBP2**	NM_001296.3	ACKR2, CMKBR9, CCR10, D6, CCR9
**CCL11**	NM_002986.2	SCYA11, eotaxin, MGC22554
**CCL13**	NM_005408.2	SCYA13, MCP-4, NCC-1, SCYL1, CKb10, MGC17134
**CCL15**	NM_032965.3	HCC-2, NCC-3, SCYL3, MIP-5, Lkn-1, MIP-1d, HMRP-2B
**CCL16**	NM_004590.2	SCYA16, NCC-4, SCYL4, LEC, HCC-4, LMC, LCC-1, CKb12, Mtn-1
**CCL18**	NM_002988.2	SCYA18, DC-CK1, PARC, AMAC-1, DCCK1, MIP-4, CKb7
**CCL19**	NM_006274.2	SCYA19, ELC, MIP-3b, exodus-3, CKb11
**CCL2**	NM_002982.3	SCYA2, MCP1, MCP-1, MCAF, SMC-CF, GDCF-2, HC11, MGC9434
**CCL20**	NM_004591.1	SCYA20, LARC, MIP-3a, exodus-1, ST38, CKb4
**CCL22**	NM_002990.3	SCYA22, MDC, STCP-1, ABCD-1, DC/B-CK, A-152E5.1, MGC34554
**CCL23**	NM_145898.1	SCYA23, Ckb-8, MPIF-1, MIP-3, CKb8
**CCL24**	NM_002991.2	SCYA24, Ckb-6, MPIF-2, eotaxin-2, MPIF2
**CCL26**	NM_006072.4	SCYA26, MIP-4alpha, eotaxin-3, IMAC, MIP-4a, TSC-1
**CCL3**	NM_002983.2	G0S19-1, LD78ALPHA, MIP-1-alpha
**CCL4**	NM_002984.2	MIP-1-beta, Act-2, AT744.1
**CCL5**	NM_002985.2	D17S136E, SCYA5, RANTES, SISd, TCP228, MGC17164
**CCL7**	NM_006273.2	SCYA6, SCYA7, MCP-3, NC28, FIC, MARC, MCP3
**CCL8**	NM_005623.2	SCYA8, MCP-2, HC14
**CCND3**	NM_001760.2	
**CCR1**	NM_001295.2	SCYAR1, CMKBR1, CKR-1, MIP1aR, CD191
**CCR10**	NM_016602.2	GPR2
**CCR2**	NM_001123041.2	CMKBR2, CC-CKR-2, CKR2, MCP-1-R, CD192, FLJ78302
**CCR5**	NM_000579.1	CMKBR5, CKR-5, CC-CKR-5, CKR5, CD195, IDDM22
**CCR6**	NM_031409.2	STRL22, CKR-L3, GPR-CY4, CMKBR6, GPR29, DRY-6, DCR2, BN-1, CD196
**CCR7**	NM_001838.2	CMKBR7, EBI1, BLR2, CDw197, CD197
**CCR8**	NM_005201.2	CMKBRL2, CMKBR8, CY6, TER1, CKR-L1, GPR-CY6, CDw198
**CCRL1**	NM_016557.2	ACKR4, CCR11, CCBP2, VSHK1, CCX-CKR, PPR1
**CCRL2**	NM_003965.4	HCR, CRAM-B, CKRX, CRAM-A

**CD14**	NM_000591.2	
**CD160**	NM_007053.2	BY55, NK1, NK28
**CD163**	NM_004244.4	M130, MM130
**CD164**	NM_006016.4	MUC-24, MGC-24
**CD19**	NM_001770.4	
**CD1A**	NM_001763.2	CD1
**CD1D**	NM_001766.3	
**CD2**	NM_001767.3	SRBC
**CD209**	NM_021155.2	DC-SIGN, CDSIGN, DC-SIGN1, CLEC4L
**CD22**	NM_001771.2	SIGLEC-2, SIGLEC2
**CD24**	NM_013230.2	CD24A
**CD244**	NM_016382.2	2B4, NAIL, NKR2B4, Nmrk, SLAMF4
**CD247**	NM_198053.1	CD3Z, CD3H, CD3Q
**CD27**	NM_001242.4	TNFRSF7, S152, Tp55
**CD274**	NM_014143.3	PDCD1LG1, B7-H, B7H1, PD-L1, PDL1, B7-H1
**CD276**	NM_001024736.1	B7-H3, B7H3
**CD28**	NM_001243078.1	
**CD34**	NM_001025109.1	
**CD36**	NM_001001548.2	SCARB3, GPIV, FAT, GP4, GP3B
**CD3D**	NM_000732.4	T3D
**CD3E**	NM_000733.2	
**CD3EAP**	NM_012099.1	POLR1G, ASE-1, CAST, PAF49
**CD4**	NM_000616.4	
**CD40**	NM_001250.4	TNFRSF5, p50, Bp50
**CD40LG**	NM_000074.2	HIGM1, IMD3, TNFSF5, CD40L, TRAP, gp39, hCD40L, CD154
**CD44**	NM_001001392.1	MIC4, MDU2, MDU3, IN, MC56, Pgp1, CD44R, HCELL, CSPG8
**CD46**	NM_172350.1	MIC10, MCP, TRA2.10, MGC26544, TLX
**CD48**	NM_001778.2	BCM1, BLAST, mCD48, hCD48, SLAMF2
**CD5**	NM_014207.2	LEU1, T1
**CD53**	NM_001040033.1	MOX44, TSPAN25
**CD55**	NM_000574.3	DAF, CR, TC, CROM
**CD58**	NM_001779.2	LFA3
**CD59**	NM_000611.4	MIC11, MIN1, MSK21, MIN2, MIN3, 16.3A5, EJ16, EJ30, EL32, G344, p18-20
**CD6**	NM_006725.3	Tp120
**CD7**	NM_006137.6	GP40, LEU-9, TP41, Tp40
**CD70**	NM_001252.2	CD27LG, TNFSF7, CD27L
**CD74**	NM_001025159.1	DHLAG
**CD79A**	NM_001783.3	IGA, MB-1
**CD79B**	NM_021602.2	IGB, B29
**CD80**	NM_005191.3	CD28LG, CD28LG1, B7.1, B7-1
**CD81**	NM_004356.3	TAPA1, TAPA-1, TSPAN28
**CD82**	NM_002231.3	ST6, KAI1, R2, IA4, TSPAN27
**CD83**	NM_004233.3	HB15, BL11
**CD86**	NM_175862.3	CD28LG2, B7.2, B7-2
**CD8A**	NM_001768.5	CD8

**CD8B**	NM_004931.3	CD8B1
**CD9**	NM_001769.2	MIC3, BA2, P24, TSPAN29, MRP-1
**CD96**	NM_005816.4	TACTILE
**CD97**	NM_078481.2	ADGRE5, TM7LN1
**CD99**	NM_002414.3	MIC2
**CDH5**	NM_001795.3	7B4, CD144
**CDKN1A**	NM_000389.2	CDKN1, P21, CIP1, WAF1, SDI1, CAP20, p21CIP1, p21Cip1/Waf1
**CEACAM1**	NM_001712.3	BGP, BGP1, CD66a
**CEACAM6**	NM_002483.4	NCA, CD66c
**CEACAM8**	NM_001816.3	CGM6, CD66b
**CEBPB**	NM_005194.2	TCF5, LAP, CRP2, NFIL6, IL6DBP, C/EBP-beta
**CFB**	NM_001710.5	BFD, BF, H2-Bf
**CFD**	NM_001928.2	DF, PFD, ADN
**CFH**	NM_001014975.2	HF, HF1, HF2, HUS, FHL1, ARMS1
**CFI**	NM_000204.3	IF, FI, C3b-INA, KAF
**CFP**	NM_002621.2	PFC
**CHUK**	NM_001278.3	TCF16, IKK1, IKK-alpha, IkBKA, NFKBIKA, IKKA
**CIITA**	NM_000246.3	MHC2TA, C2TA, NLRA
**CISH**	NM_145071.2	CIS, G18, CIS-1, SOCS
**CLEC4A**	NM_194448.2	CLECSF6, DCIR, DDB27
**CLEC4E**	NM_014358.2	CLECSF9, mincle
**CLEC5A**	NM_013252.2	CLECSF5, MDL-1
**CLEC6A**	NM_001007033.1	CLECSF10, dectin-2
**CLEC7A**	NM_197954.2	CLECSF12, dectin-1, hDectin-1
**CLU**	NM_001831.2	CLI, APOJ, SGP-2, SP-40, TRPM-2, KUB1
**CMKLR1**	NM_004072.1	
**CR1**	NM_000651.4	CD35, KN
**CR2**	NM_001006658.1	CD21
**CRADD**	NM_003805.3	RAIDD
**CSF1**	NM_000757.4	M-CSF, MCSF, MGC31930
**CSF1R**	NM_005211.2	FMS, C-FMS, CSFR, CD115
**CSF2**	NM_000758.2	GM-CSF, GMCSF
**CSF2RB**	NM_000395.2	IL3RB, IL5RB, CD131
**CSF3R**	NM_156038.2	CD114, GCSFR
**CTLA4_all**	NM_005214.3	CELIAC3, CD152, CD, GSE, CD28, ICOS
**CTLA4-TM**	NM_005214.3	CELIAC3, CD152, CD, GSE, CD28, ICOS
**sCTLA4**	NM_001037631.1	CELIAC3, CD152, CD, GSE, CD28, ICOS
**CTNNB1**	NM_001098210.1	CTNNB, beta-catenin
**CTSC**	NM_001814.4	PLS, PALS, DPP1
**CTSG**	NM_001911.2	CG
**CTSS**	NM_004079.3	
**CUL9**	NM_015089.2	H7AP1, KIAA0708, PARC
**CX3CL1**	NM_002996.3	SCYD1, NTN, C3Xkine, ABCD-3, CXC3C, CXC3, fractalkine, neurotactin
**CX3CR1**	NM_001337.3	GPR13, CMKBRL1, CMKDR1, V28, CCRL1
**CXCL1**	NM_001511.1	MGSA, GRO1, FSP, SCYB1, GROa, MGSA-a, NAP-3
**CXCL10**	NM_001565.1	INP10, SCYB10, IFI10, IP-10, crg-2, mob-1, C7, gIP-10

**CXCL11**	NM_005409.4	SCYB9B, SCYB11, H174, b-R1, I-TAC, IP-9
**CXCL12**	NM_000609.5	SDF1A, SDF1B, SDF1, SCYB12, SDF-1a, SDF-1b, PBSF, TLSF-a, TLSF-b, TPAR1
**CXCL13**	NM_006419.2	SCYB13, BLC, BCA-1, BLR1L, ANGIE, ANGIE2
**CXCL2**	NM_002089.3	GRO2, SCYB2, GROb, MIP-2a, MGSA-b, CINC-2a
**CXCL9**	NM_002416.1	CMK, MIG, SCYB9, Humig, crg-10
**CXCR1**	NM_000634.2	CMKAR1, IL8RA, CKR-1, CDw128a, CD181
**CXCR2**	NM_001557.2	IL8RB, CMKAR2, CD182
**CXCR3**	NM_001504.1	GPR9, CKR-L2, CMKAR3, IP10-R, MigR, CD183
**CXCR4**	NM_003467.2	LESTR, NPY3R, HM89, NPYY3R, D2S201E, fusin, HSY3RR, NPYR, CD184
**CXCR6**	NM_006564.1	TYMSTR, STRL33, BONZO, CD186
**CYBB**	NM_000397.3	CGD, GP91-PHOX, NOX2
**DEFB1**	NM_005218.3	HBD-1, DEFB-1, DEFB101, HBD1
**DEFB103A**	NM_001081551.2	HBD-3, HBP-3, HBD3, HBP3, DEFB-3
**DEFB103B**	NM_018661.3	
**DEFB4A**	NM_004942.2	SAP1, HBD-2, DEFB-2
**DPP4**	NM_001935.3	CD26, ADCP2, DPPIV
**DUSP4**	NM_057158.2	HVH2, MKP-2, TYP
**EBI3**	NM_005755.2	
**EDNRB**	NM_003991.2	HSCR2, HSCR, ETB
**EGR1**	NM_001964.2	TIS8, G0S30, NGFI-A, KROX-24, ZIF-268, AT225, ZNF225
**EGR2**	NM_000399.3	KROX20
**ENTPD1**	NM_001098175.1	CD39, NTPDase-1, ATPDase
**EOMES**	NM_005442.2	TBR2
**ETS1**	NM_005238.3	EWSR2, FLJ10768, ETS-1
**FADD**	NM_003824.2	MORT1, GIG3
**FAS**	NM_000043.3	FAS1, APT1, TNFRSF6, CD95, APO-1
**FCAR**	NM_133280.1	CD89
**FCER1A**	NM_002001.2	FCE1A
**FCER1G**	NM_004106.1	
**FCGR1A/B**	NM_000566.3	CD64, CD64A/CD64B
**FCGR2A**	NM_021642.3	FCG2, FCGR2A1, FCGR2, CD32, CD32A, IGFR2, CDw32
**FCGR2A/C**	NM_201563.4	FCG2, FCGR2A1, FCGR2, CD32, CD32A, IGFR2, CDw32/hFcRII-C, CD32C
**FCGR2B**	NM_001002273.1	FCG2, FCGR2, CD32, CD32B
**FCGR3A/B**	NM_000570.4	CD16, CD16a/CD16b
**FCGRT**	NM_004107.4	FCRN, alpha-chain
**FKBP5**	NM_001145775.1	FKBP51, FKBP54, PPIase, P54, Ptg-10
**FN1**	NM_212482.1	MSF, CIG, LETS, GFND2, FINC
**FOXP3**	NM_014009.3	IPEX, JM2, XPID, AIID, PIDX, DIETER, SCURFIN
**FYN**	NM_002037.3	SYN, SLK, MGC45350
**GATA3**	NM_001002295.1	HDR
**GBP1**	NM_002053.1	
**GBP5**	NM_052942.3	
**GFI1**	NM_005263.2	ZNF163
**GNLY**	NM_006433.2	LAG2, NKG5, LAG-2, D2S69E, TLA519
**GP1BB**	NM_000407.4	CD42c

**GPI**	NM_000175.2	AMF, NLK
**GPR183**	NM_004951.3	EBI2
**GZMA**	NM_006144.2	HFSP, CTLA3
**GZMB**	NM_004131.3	CTLA1, CSPB, CCPI, CGL-1, CSP-B, CGL1, CTSGL1, HLP, SECT
**GZMK**	NM_002104.2	TRYP2, PRSS
**HAMP**	NM_021175.2	LEAP-1, HEPC, HFE2B, LEAP1
**HAVCR2**	NM_032782.3	Tim-3, TIM3, FLJ14428, TIMD3
**HFE**	NM_139011.2	HLA-H
**HLA-A**	NM_002116.5	
**HLA-B**	NM_005514.6	
**HLA-C**	NM_002117.4	
**HLA-DMA**	NM_006120.3	D6S222E, RING6
**HLA-DMB**	NM_002118.3	D6S221E, RING7
**HLA-DOB**	NM_002120.3	
**HLA-DPA1**	NM_033554.2	HLA-DP1A
**HLA-DPB1**	NM_002121.4	HLA-DP1B
**HLA-DQA1**	NM_002122.3	CELIAC1
**HLA-DQB1**	NM_002123.3	HLA-DQB, IDDM1, CELIAC1
**HLA-DRA**	NM_019111.3	HLA-DRA1
**HLA-DRB1**	NM_002124.2	
**HLA-DRB3**	NM_022555.3	
**HRAS**	NM_005343.2	HRAS1
**ICAM1**	NM_000201.2	BB2, CD54
**ICAM2**	NM_000873.3	CD102
**ICAM3**	NM_002162.3	CDW50, ICAM-R, CD50
**ICAM4**	NM_001039132.1	LW, CD242
**ICAM5**	NM_003259.3	TLCN, TLN
**ICOS**	NM_012092.2	AILIM, CD278
**ICOSLG**	NM_015259.4	ICOSL, KIAA0653, GL50, B7-H2, B7RP-1, B7H2, B7RP1, ICOS-L, CD275
**IDO1**	NM_002164.3	IDO, INDO
**IFI16**	NM_005531.1	IFNGIP1, PYHIN2
**IFI35**	NM_005533.3	IFP35
**IFIH1**	NM_022168.2	MDA-5, Hlcd, MDA5, IDDM19
**IFIT2**	NM_001547.4	IFI54, G10P2, IFI-54, ISG-54K, cig42, GARG-39
**IFITM1**	NM_003641.3	IFI17, 9-27, CD225
**IFNA1/13**	NM_024013.1	IFNA@, IFL, IFN, IFN-ALPHA, IFNA13, IFN-alphaD
**IFNA2**	NM_000605.3	IFNA, IFN-alphaA
**IFNAR1**	NM_000629.2	IFNAR, IFRC
**IFNAR2**	NM_000874.3	IFNABR
**IFNB1**	NM_002176.2	IFNB, IFB, IFF
**IFNG**	NM_000619.2	
**IFNGR1**	NM_000416.1	IFNGR, CD119
**IGF2R**	NM_000876.1	CD222, MPRI, MPR1, CIMPR, M6P-R
**IKBKAP**	NM_003640.3	DYS, IKAP, TOT1, ELP1, IKI3
**IKBKB**	NM_001556.1	IKK2, NFKBIKB, IKK-beta, IKKB
**IKBKE**	NM_014002.2	IKKE, IKK-i, KIAA0151

**IKBKG**	NM_003639.2	IP2, IP1, IKK-gamma, NEMO, Fip3p, FIP-3, FIP3
**IKZF1**	NM_006060.3	ZNFN1A1, hIk-1, LyF-1, Hs.54452, IKAROS
**IKZF2**	NM_016260.2	ZNFN1A2, Helios
**IKZF3**	NM_183232.2	ZNFN1A3, Aiolos
**IL10**	NM_000572.2	CSIF, TGIF, IL10A, IL-10
**IL10RA**	NM_001558.2	IL10R, HIL-10R, CDW210A, CD210a
**IL11RA**	NM_147162.1	
**IL12A**	NM_000882.2	NKSF1, CLMF, IL-12A, p35, NFSK
**IL12B**	NM_002187.2	NKSF2, CLMF, IL-12B, NKSF, CLMF2
**IL12RB1**	NM_005535.1	IL12RB, CD212
**IL13**	NM_002188.2	P600, IL-13, ALRH, BHR1, MGC116786, MGC116788, MGC116789
**IL13RA1**	NM_001560.2	IL-13Ra, NR4, CD213a1
**IL15**	NM_172174.1	IL-15, MGC9721
**IL16**	NM_004513.4	LCF, IL-16, prIL-16, HsT19289, FLJ42735, FLJ16806
**IL17A**	NM_002190.2	CTLA8, IL17, IL-17A, IL-17
**IL17B**	NM_014443.2	IL-17B, ZCYTO7, IL-20, MGC138900, MGC138901, NIRF
**IL17F**	NM_052872.3	IL-17F, ML-1, ML1
**IL18**	NM_001562.2	IGIF, IL1F4, IL-1g, IL-18
**IL18R1**	NM_003855.2	IL1RRP, IL-1Rrp, CD218a
**IL18RAP**	NM_003853.2	AcPL, CD218b
**IL19**	NM_013371.3	IL-19, MDA1, ZMDA1, IL-10C, NG.1
**IL1A**	NM_000575.3	IL1, IL1F1, IL-1A, IL1-ALPHA
**IL1B**	NM_000576.2	IL1F2, IL-1B, IL1-BETA
**IL1R1**	NM_000877.2	IL1R, IL1RA, D2S1473, CD121A
**IL1R2**	NM_173343.1	IL1RB, CD121b
**IL1RAP**	NM_002182.2	IL-1RAcP, IL1R3, C3orf13
**IL1RL1**	NM_016232.4	ST2, FIT-1, ST2L, ST2V, DER4, T1, IL33R
**IL1RL2**	NM_003854.2	IL1R-rp2, IL1RRP2
**IL1RN**	NM_000577.3	IL1RA, ICIL-1RA, IL1F3, IRAP, IL-1RN, MGC10430
**IL2**	NM_000586.2	IL-2, TCGF
**IL20**	NM_018724.3	ZCYTO10, IL10D, IL-20
**IL21**	NM_021803.2	Za11, IL-21
**IL21R**	NM_021798.2	CD360
**IL22**	NM_020525.4	ILTIF, IL-21, zcyto18, IL-TIF, IL-D110, TIFa, TIFIL-23, IL-22, MGC79382, MGC79384
**IL22RA2**	NM_181310.1	CRF2-S1, IL-22BP
**IL23A**	NM_016584.2	SGRF, IL23P19, IL-23, IL-23A, P19
**IL23R**	NM_144701.2	IL-23R
**IL26**	NM_018402.1	AK155, IL-26
**IL27**	NM_145659.3	IL30, IL-27, p28, IL27p28, IL-27A, IL27A, MGC71873
**IL28A**	NM_172138.1	IL-28A, IFNL2
**IL28A/B**	NM_172139.2	IL-28A, IFNL2/IL-28B, IFNL3, IL28C
**IL29**	NM_172140.1	IL-29, IFNL1
**IL2RA**	NM_000417.1	IL2R, CD25
**IL2RB**	NM_000878.2	IL15RB, CD122
**IL2RG**	NM_000206.1	SCIDX1, IMD4, CIDX, CD132

**IL3**	NM_000588.3	IL-3, MULTI-CSF, MCGF, MGC79398, MGC79399
**IL32**	NM_001012633.1	NK4, TAIF, TAIFb, TAIFd
**IL4**	NM_000589.2	BSF1, IL-4, BCGF1, BCGF-1, MGC79402
**IL4R**	NM_000418.2	CD124
**IL5**	NM_000879.2	IL-5, EDF, TRF
**IL6**	NM_000600.1	IFNB2, IL-6, BSF2, HGF, HSF
**IL6R**	NM_000565.2	CD126
**IL6ST**	NM_002184.2	GP130, CD130
**IL7**	NM_000880.2	IL-7
**IL7R**	NM_002185.2	CD127
**IL8**	NM_000584.2	SCYB8, LUCT, LECT, MDNCF, TSG-1, CXCL8, IL-8, NAP-1, 3-10C, MONAP, AMCF-I, LYNAP, NAF, b-ENAP, GCP-1, K60, GCP1, NAP1
**IL9**	NM_000590.1	IL-9, HP40, P40
**ILF3**	NM_001137673.1	NF90, MPHOSPH4, MPP4, DRBP76, NFAR-1
**IRAK1**	NM_001569.3	IRAK, pelle
**IRAK2**	NM_001570.3	
**IRAK3**	NM_007199.1	IRAK-M
**IRAK4**	NM_016123.1	NY-REN-64
**IRF1**	NM_002198.1	MAR
**IRF3**	NM_001571.5	
**IRF4**	NM_002460.1	MUM1, LSIRF
**IRF5**	NM_002200.3	
**IRF7**	NM_001572.3	
**IRF8**	NM_002163.2	ICSBP1, IRF-8, ICSBP
**IRGM**	NM_001145805.1	IRGM1, LRG47, LRG-47, IFI1
**ITGA2B**	NM_000419.3	GP2B, CD41B, CD41
**ITGA4**	NM_000885.4	CD49D, CD49d
**ITGA5**	NM_002205.2	FNRA, CD49e
**ITGA6**	NM_000210.1	CD49f
**ITGAE**	NM_002208.4	CD103, HUMINAE
**ITGAL**	NM_002209.2	CD11A, LFA-1
**ITGAM**	NM_000632.3	CR3A, CD11B, MAC-1, CD11b
**ITGAX**	NM_000887.3	CD11C, CD11c
**ITGB1**	NM_033666.2	FNRB, MSK12, MDF2, CD29, GPIIA
**ITGB2**	NM_000211.2	CD18, MFI7, LFA-1, MAC-1
**ITLN1**	NM_017625.2	ITLN, FLJ20022, LFR, HL-1, hIntL
**ITLN2**	NM_080878.2	HL-2
**JAK1**	NM_002227.1	JAK1B, JAK1A, JTK3
**JAK2**	NM_004972.2	JTK10
**JAK3**	NM_000215.2	L-JAK, JAKL, LJAK, JAK3_HUMAN, JAK-3
**KCNJ2**	NM_000891.2	Kir2.1, IRK1
**KIR_Activating_Subgroup_1**	NM_001083539.1	KIR3DS1,KIR2DS1,KIR2DS2,KIR2DS4
**KIR_Activating_Subgroup_2**	NM_014512.1	KIR2DS1,KIR2DS2,KIR2DS3,KIR2DS4&KIR2DS5
**KIR_Inhibiting_Subgroup_1**	NM_014218.2	KIR2DL1,KIR2DL2,KIR2DL4,KIR2DL5,KIR3DL1&KIR3DL3
**KIR_Inhibiting_Subgroup_2**	NM_014511.3	KIR2DL3,KIR2DL4,KIR2DL5&KIR3DL3,KIR2DL1,KIR2DL2,KIR3DL1,KIR3DL2

**KIR3DL1**	NM_013289.2	cl-2, NKB1, cl-11, nkat3, NKB1B, AMB11, CD158e1/2, CD158e1, CD158e2
**KIR3DL2**	NM_006737.2	cl-5, nkat4, nkat4a, nkat4b, CD158K
**KIR3DL3**	NM_153443.3	KIRC1, KIR3DL7, KIR44, CD158z
**KIT**	NM_000222.2	PBT, CD117, SCFR, C-Kit
**KLRAP1**	NR_028045.1	KLRA1, Ly49, LY49L
**KLRB1**	NM_002258.2	NKR, CD161, NKR-P1, NKR-P1A, hNKR-P1A, CLEC5B
**KLRC1**	NM_002259.3	NKG2, NKG2-A, NKG2-B, CD159a
**KLRC2**	NM_002260.3	NKG2-C, CD159c
**KLRC3**	NM_007333.2	NKG2-E
**KLRC4**	NM_013431.2	NKG2-F
**KLRD1**	NM_002262.3	CD94
**KLRF1**	NM_016523.1	CLEC5C, NKp80
**KLRF2**	NM_001190765.1	NKp65
**KLRG1**	NM_005810.3	MAFA, 2F1, MAFA-L, CLEC15A
**KLRG2**	NM_198508.2	FLJ44186, CLEC15B
**KLRK1**	NM_007360.1	NKG2D, KLR, NKG2-D, CD314
**LAG3**	NM_002286.5	CD223
**LAIR1**	NM_002287.3	CD305
**LAMP3**	NM_014398.3	LAMP, TSC403, DC-LAMP, DCLAMP, CD208
**LCK**	NM_005356.2	
**LCP2**	NM_005565.3	SLP76, SLP-76
**LEF1**	NM_016269.3	TCF1ALPHA, TCF10, TCF7L3
**LGALS3**	NM_001177388.1	LGALS2, MAC-2, GALIG
**LIF**	NM_002309.3	CDF, DIA, HILDA
**LILRA1**	NM_006863.1	LIR-6, CD85i, LIR6
**LILRA2**	NM_006866.2	LIR-7, ILT1, CD85h, LIR7
**LILRA3**	NM_006865.3	LIR-4, HM43, ILT6, HM31, LIR4, CD85e
**LILRA4**	NM_012276.3	ILT7, CD85g
**LILRA5**	NM_181879.2	LILRB7, ILT11, LIR9, CD85, CD85f
**LILRA6**	NM_024318.2	LILRB6, ILT8, CD85b
**LILRB1**	NM_001081637.1	LIR-1, ILT2, MIR-7, CD85, LIR1, CD85j
**LILRB2**	NM_005874.1	LIR-2, ILT4, MIR-10, LIR2, CD85d, MIR10
**LILRB3**	NM_006864.2	LIR-3, HL9, ILT5, LIR3, CD85a
**LILRB4**	NM_001081438.1	LIR-5, ILT3, HM18, LIR5, CD85k
**LILRB5**	NM_001081442.1	LIR-8, LIR8, CD85c
**LITAF**	NM_004862.3	PIG7, SIMPLE, FLJ38636, TP53I7
**LTA**	NM_000595.2	TNFB, TNFSF1, LT
**LTB4R**	NM_181657.3	P2RY7, GPR16, CMKRL1, BLTR, P2Y7, LTB4R1
**LTB4R2**	NM_019839.4	BLTR2, BLT2, JULF2, NOP9
**LTBR**	NM_002342.1	D12S370, TNFCR, TNFR-RP, TNFR2-RP, TNF-R-III, TNFRSF3
**LTF**	NM_002343.2	HLF2
**LY96**	NM_015364.2	MD-2
**MAF**	NM_005360.4	c-MAF
**MALT1**	NM_006785.2	MLT
**MAP4K1**	NM_007181.3	HPK1

**MAP4K2**	NM_004579.2	RAB8IP, GCK, BL44
**MAP4K4**	NM_004834.3	HGK, NIK, FLH21957
**MAPK1**	NM_138957.2	PRKM2, PRKM1, ERK, ERK2, p41mapk, MAPK2
**MAPK11**	NM_002751.5	PRKM11, p38-2, p38Beta, SAPK2
**MAPK14**	NM_001315.1	CSPB1, CSBP1, CSBP2, PRKM14, p38, Mxi2, PRKM15
**MAPKAPK2**	NM_004759.3	
**MARCO**	NM_006770.3	SCARA2
**MASP1**	NM_139125.3	CRARF, PRSS5, MASP
**MASP2**	NM_139208.1	MASP1P1
**MBL2**	NM_000242.2	MBL, COLEC1
**MBP**	NM_002385.2	
**MCL1**	NM_021960.3	BCL2L3, Mcl-1
**MIF**	NM_002415.1	GIF
**MME**	NM_000902.2	CALLA, CD10, NEP
**MR1**	NM_001531.2	HLALS
**MRC1**	NM_002438.2	CLEC13D, CD206, bA541I19.1, CLEC13DL
**MS4A1**	NM_152866.2	CD20, B1, Bp35, MS4A2
**MSR1**	NM_002445.3	SCARA1, CD204
**MUC1**	NM_001018017.1	PUM, CD227, PEM
**MX1**	NM_002462.2	IFI-78K, MxA
**MYD88**	NM_002468.3	
**NCAM1**	NM_000615.5	NCAM, CD56
**NCF4**	NM_000631.4	p40phox, SH3PXD4
**NCR1**	NM_004829.5	LY94, NK-p46, NKP46, CD335
**NFATC1**	NM_172389.1	NF-ATC, NFATc, NFAT2
**NFATC2**	NM_012340.3	NF-ATP, NFATp, NFAT1
**NFATC3**	NM_004555.2	NFAT4, NFATX
**NFIL3**	NM_005384.2	IL3BP1, E4BP4, NFIL3A, NF-IL3A
**NFKB1**	NM_003998.2	KBF1, p105, NFKB-p50, p50, NF-kappaB, NFkappaB
**NFKB2**	NM_002502.2	LYT-10, p52
**NFKBIA**	NM_020529.1	NFKBI, IKBA, MAD-3, IkappaBalpha
**NFKBIZ**	NM_001005474.1	MAIL, FLJ34463
**NLRP3**	NM_001079821.2	C1orf7, CIAS1, AGTAVPRL, AII, AVP, FCAS, FCU, NALP3, PYPAF1, MWS, CLR1.1
**NOD1**	NM_006092.1	CARD4, NLRC1, CLR7.1
**NOD2**	NM_022162.1	IBD1, CARD15, BLAU, CD, PSORAS1, CLR16.3, NLRC2
**NOS2**	NM_000625.4	NOS2A, iNOS, NOS, HEP-NOS
**NOTCH1**	NM_017617.3	TAN1
**NOTCH2**	NM_024408.3	
**NT5E**	NM_002526.2	NT5, CD73, eN, eNT
**PAX5**	NM_016734.1	BSAP
**PDCD1**	NM_005018.1	CD279, PD1
**PDCD1LG2**	NM_025239.3	PD-L2, Btdc, PDL2, bA574F11.2, CD273, B7-DC
**PDCD2**	NM_144781.2	ZMYND7, RP8
**PDGFB**	NM_033016.2	SIS, SSV
**PDGFRB**	NM_002609.3	PDGFR, JTK12, CD140b, PDGFR1

**PECAM1**	NM_000442.3	CD31
**PIGR**	NM_002644.2	
**PLA2G2A**	NM_000300.2	PLA2B, PLA2L
**PLA2G2E**	NM_014589.1	
**PLAU**	NM_002658.2	URK, UPA
**PLAUR**	NM_001005376.1	URKR, UPAR, CD87
**PML**	NM_002675.3	MYL, TRIM19, RNF71
**POU2F2**	NM_002698.2	OTF2, 40818
**PPARG**	NM_015869.3	PPARG1, PPARG2, NR1C3, PPARgamma
**PPBP**	NM_002704.2	THBGB1, SCYB7, TGB, NAP-2-L1, LA-PF4, MDGF, LDGF, Beta-TG, CTAP3, CXCL7, PBP, b-TG1, TGB1, CTAPIII, NAP-2
**PRDM1**	NM_001198.3	BLIMP1, PRDI-BF1
**PRF1**	NM_005041.3	PFP, P1, HPLH2
**PRKCD**	NM_006254.3	
**PSMB10**	NM_002801.2	MECL1, LMP10, MGC1665, beta2i
**PSMB5**	NM_001130725.1	X, MB1
**PSMB7**	NM_002799.2	Z
**PSMB8**	NM_004159.4	LMP7, RING10, D6S216E, PSMB5i, beta5i
**PSMB9**	NM_002800.4	LMP2, RING12, beta1i, PSMB6i
**PSMC2**	NM_002803.3	MSS1, S7, Nbla10058
**PSMD7**	NM_002811.3	S12, P40, MOV34, Rpn8
**PTAFR**	NM_000952.3	
**PTGER4**	NM_000958.2	EP4
**PTGS2**	NM_000963.1	COX2
**PTK2**	NM_005607.3	FAK, FADK, FAK1
**PTPN2**	NM_002828.2	PTPT, TCELLPTP, TC-PTP, TCPTP
**PTPN22**	NM_015967.4	PTPN8, Lyp, Lyp1, Lyp2
**PTPN6**	NM_002831.5	HCP, HCPH, PTP-1C, SHP-1, SHP1
**PTPRC_all**	NM_080921.2	CD45, LCA, T200, GP180
**CD45R0**	NM_080921.3	CD45, LCA, T200, GP180
**CD45RA**	NM_002838.4	CD45, LCA, T200, GP180
**CD45RB**	ENST00000367367.1	CD45, LCA, T200, GP180
**PYCARD**	NM_013258.3	TMS-1, CARD5, ASC
**RAF1**	NM_002880.2	Raf-1, c-Raf, CRAF
**RAG1**	NM_000448.2	RNF74, MGC43321
**RAG2**	NM_000536.3	
**RARRES3**	NM_004585.3	PLAAT4, TIG3, HRASLS4
**RELA**	NM_021975.2	NFKB3, p65
**RELB**	NM_006509.2	REL-B
**RORC**	NM_001001523.1	RZRG, RORG, NR1F3, TOR
**RUNX1**	NM_001754.4	AML1, CBFA2, PEBP2A2, AMLCR1
**S100A8**	NM_002964.3	CAGA, CFAG, P8, MRP8, 60B8AG, CGLA
**S100A9**	NM_002965.2	P14, MIF, NIF, LIAG, MRP14, MAC387, 60B8AG, CGLB
**S1PR1**	NM_001400.3	EDG1, edg-1, D1S3362, CD363
**SELE**	NM_000450.2	ELAM1, ELAM, ESEL, CD62E

**SELL**	NR_029467.1	LYAM1, LNHR, LSEL, LAM1, LAM-1, hLHRc, Leu-8, Lyam-1, PLNHR, CD62L
**SELPLG**	NM_003006.3	PSGL-1, CD162
**SERPING1**	NM_000062.2	C1NH, C1IN, C1-INH, HAE1, HAE2
**SH2D1A**	NM_001114937.2	IMD5, LYP, XLP, MTCP1, DSHP, XLPD, EBVS, SAP
**SIGIRR**	NM_021805.2	TIR8
**SKI**	NM_003036.2	
**SLAMF1**	NM_003037.2	SLAM, CD150
**SLAMF6**	NM_001184714.1	KALI, NTBA, KALIb, Ly108, SF2000, NTB-A, CD352
**SLAMF7**	NM_021181.3	CRACC, 19A, CS1, CD319
**SLC2A1**	NM_006516.2	GLUT1, GLUT, DYT18
**SMAD3**	NM_005902.3	MADH3, JV15-2, HsT17436
**SMAD5**	NM_005903.5	MADH5, Dwfc, JV5-1
**SOCS1**	NM_003745.1	SOCS-1, SSI-1, JAB, TIP3, Cish1
**SOCS3**	NM_003955.3	SSI-3, CIS3, SOCS-3, Cish3
**SPP1**	NM_000582.2	BNSP, OPN, BSPI, ETA-1
**SRC**	NM_005417.3	SRC1, ASV, c-src
**STAT1**	NM_007315.2	STAT91, ISGF-3
**STAT2**	NM_005419.2	STAT113
**STAT3**	NM_139276.2	APRF
**STAT4**	NM_003151.2	
**STAT5A**	NM_003152.2	STAT5, MGF
**STAT5B**	NM_012448.3	
**STAT6**	NM_003153.3	D12S1644, IL-4-STAT
**SYK**	NM_003177.3	
**TAGAP**	NM_054114.3	FLJ32631, IDDM21, ARHGAP47
**TAL1**	NM_003189.2	TCL5, SCL, bHLHa17
**TAP1**	NM_000593.5	ABCB2, PSF1, RING4, D6S114E
**TAP2**	NM_000544.3	ABCB3, PSF2, RING11, D6S217E
**TAPBP**	NM_003190.4	TAPA
**TBK1**	NM_013254.2	NAK
**TBX21**	NM_013351.1	TBLYM, T-bet
**TCF4**	NM_003199.1	SEF2-1B, ITF2, bHLHb19, E2-2
**TCF7**	NM_003202.2	TCF-1
**TFRC**	NM_003234.1	CD71, TFR1
**TGFB1**	NM_000660.3	TGFB, DPD1, CED, TGFbeta
**TGFBI**	NM_000358.2	CSD3, LCD1, CSD1, CSD2, BIGH3, CDB1, CDGG1
**TGFBR1**	NM_004612.2	ALK-5, ACVRLK4
**TGFBR2**	NM_001024847.1	MFS2
**THY1**	NM_006288.2	CD90
**TICAM1**	NM_014261.1	TRIF, TICAM-1, MGC35334, PRVTIRB
**TIGIT**	NM_173799.2	VSIG9, VSTM3, FLJ39873, DKFZp667A205
**TIRAP**	NM_148910.2	Mal, wyatt
**TLR1**	NM_003263.3	rsc786, KIAA0012, CD281
**TLR2**	NM_003264.3	TIL4, CD282
**TLR3**	NM_003265.2	CD283

**TLR4**	NM_138554.2	hToll, CD284
**TLR5**	NM_003268.3	TIL3, SLEB1, FLJ10052, MGC126430, MGC126431
**TLR7**	NM_016562.3	
**TLR8**	NM_016610.2	CD288
**TLR9**	NM_017442.2	CD289
**TMEM173**	NM_198282.1	STING1, FLJ38577, NET23
**TNF**	NM_000594.2	TNFA, TNFSF2, DIF, TNF-alpha
**TNFAIP3**	NM_006290.2	A20, OTUD7C
**TNFAIP6**	NM_007115.2	TSG6, TSG-6
**TNFRSF10C**	NM_003841.3	DcR1, TRAILR3, LIT, TRID, CD263
**TNFRSF11A**	NM_003839.2	RANK, CD265
**TNFRSF13B**	NM_012452.2	TACI, CD267
**TNFRSF13C**	NM_052945.3	BAFFR, CD268
**TNFRSF14**	NM_003820.2	HVEM, ATAR, TR2, LIGHTR, HVEA, CD270
**TNFRSF17**	NM_001192.2	BCMA, BCM, CD269
**TNFRSF1B**	NM_001066.2	TNFR2, TNFBR, TNFR80, TNF-R75, TNF-R-II, p75, CD120b
**TNFRSF4**	NM_003327.2	TXGP1L, ACT35, OX40, CD134
**TNFRSF8**	NM_152942.2	CD30, D1S166E, KI-1
**TNFRSF9**	NM_001561.4	ILA, CD137, 4-1BB
**TNFSF10**	NM_003810.2	TRAIL, Apo-2L, TL2, CD253
**TNFSF11**	NM_003701.2	TRANCE, RANKL, OPGL, ODF, CD254
**TNFSF12**	NM_003809.2	TWEAK, DR3LG, APO3L
**TNFSF13B**	NM_006573.4	TNFSF20, BAFF, THANK, BLYS, TALL-1, TALL1, CD257
**TNFSF15**	NM_001204344.1	TL1, VEGI, TL1A, VEGI192A, MGC129934, MGC129935
**TNFSF4**	NM_003326.2	TXGP1, OX-40L, gp34, CD252
**TNFSF8**	NM_001244.3	CD30LG, CD153
**TOLLIP**	NM_019009.2	IL-1RAcPIP
**TP53**	NM_000546.2	p53, LFS1
**TRAF1**	NM_005658.3	EBI6
**TRAF2**	NM_021138.3	TRAP3
**TRAF3**	NM_145725.1	CAP-1, CD40bp, CRAF1, LAP1
**TRAF4**	NM_004295.2	CART1, MLN62, RNF83
**TRAF5**	NM_004619.3	RNF84
**TRAF6**	NM_145803.1	RNF85
**TYK2**	NM_003331.3	JTK1
**UBE2L3**	NM_198157.1	UBCH7
**VCAM1**	NM_001078.3	CD106
**VTN**	NM_000638.3	VN
**XBP1**	NM_005080.2	XBP2
**XCL1**	NM_002995.1	LTN, SCYC1, LPTN, ATAC, SCM-1a, SCM-1, lymphotactin
**XCR1**	NM_005283.2	GPR5, CCXCR1
**ZAP70**	NM_001079.3	SRK, ZAP-70, STD
**ZBTB16**	NM_006006.4	ZNF145, PLZF
**ZEB1**	NM_001128128.1	TCF8, PPCD3, BZP, ZEB, AREB6, NIL-2-A, Zfhep, Zfhx1a
	-	
**ABCF1**	NM_001090.2	ABC50, EST123147

**ALAS1**	NM_000688.4	ALAS3, ALAS
**EEF1G**	NM_001404.4	EF1G
**G6PD**	NM_000402.2	G6PD1
**GAPDH**	NM_002046.3	GAPD
**GUSB**	NM_000181.1	
**HPRT1**	NM_000194.1	HPRT, HGPRT
**OAZ1**	NM_004152.2	OAZ, AZI, MGC138338
**POLR1B**	NM_019014.3	Rpo1-2, FLJ21921, FLJ10816, RPA2
**POLR2A**	NM_000937.2	POLR2, POLRA, RPB1
**PPIA**	NM_021130.2	CYPA
**SDHA**	NM_004168.1	FP, SDHF
**TBP**	NM_001172085.1	GTF2D1, SCA17, TFIID
**TUBB**	NM_178014.2	OK/SW-cl.56, MGC16435, M40, Tubb5
**RPL19**	NM_000981.3	FLJ27452, MGC71997, DKFZp779D216, L19
