# Supplementary figures and images for: Statins Modulate Microenvironmental Cues Driving Macrophage Polarization in Simulated Periodontal Inflammation
Source: Cells. 2023 Jul 29;12(15):1961. doi: 10.3390/cells12151961 (PMC10417531; doi:10.3390/cells12151961)

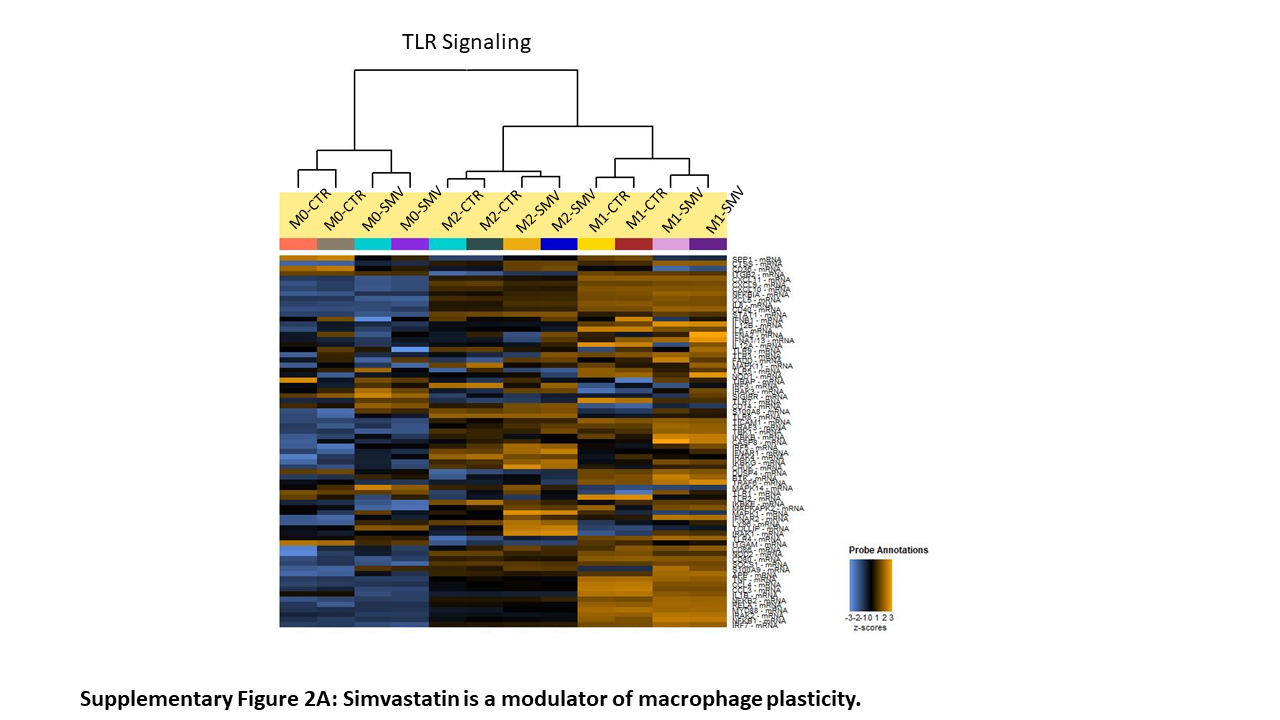

Supplement: Supplementary file 1 [file cells-12-01961-s001.zip › Supplementary Figure 2A.tif]

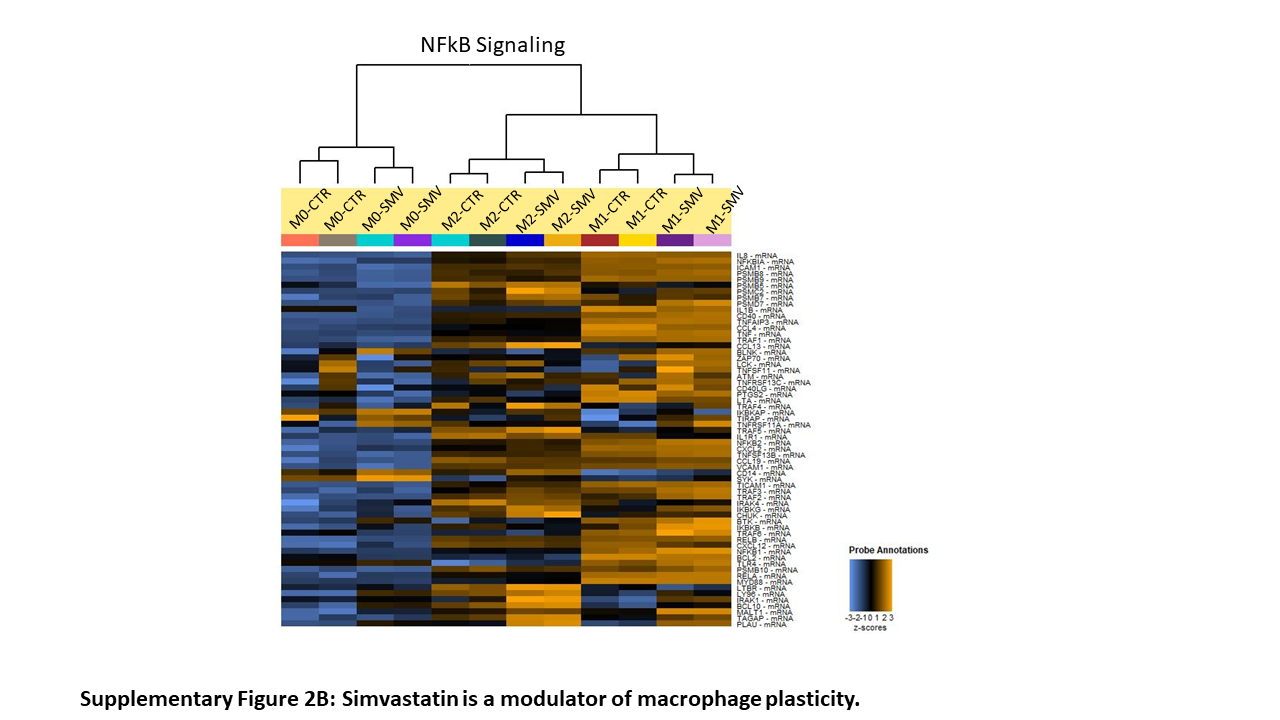

Supplement: Supplementary file 1 [file cells-12-01961-s001.zip › Supplementary Figure 2B.tif]

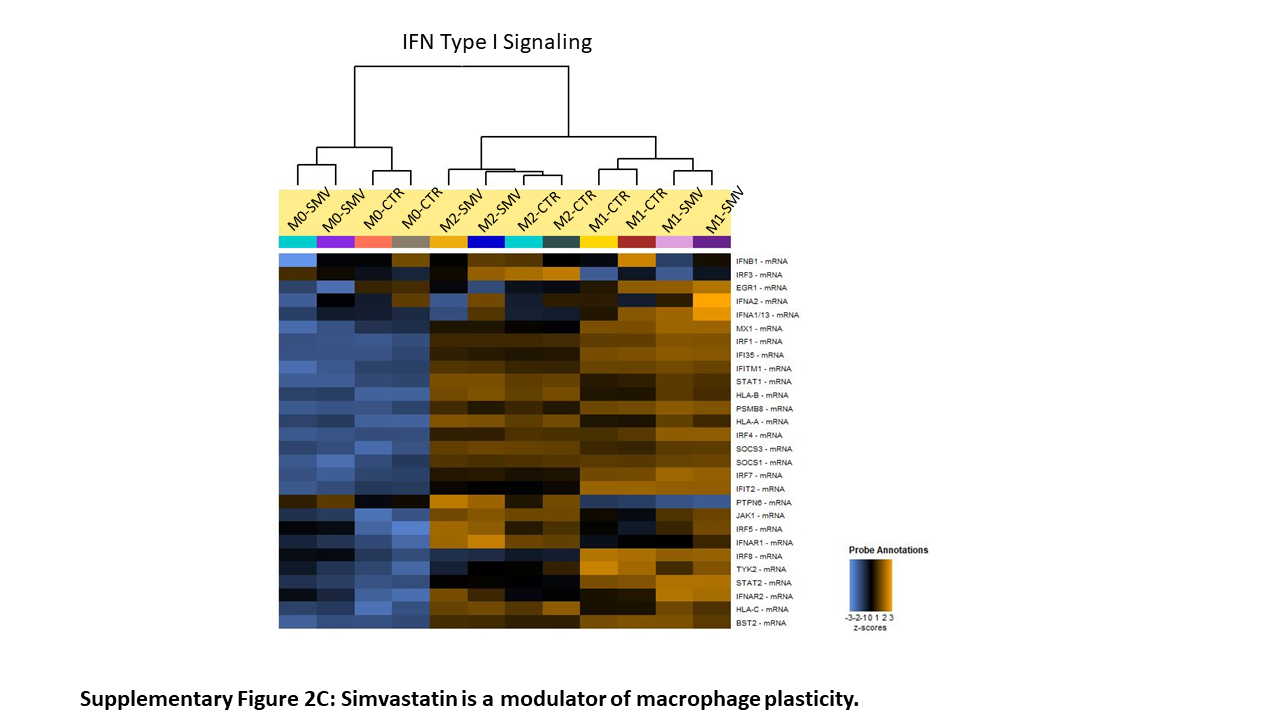

Supplement: Supplementary file 1 [file cells-12-01961-s001.zip › Supplementary Figure 2C.tif]

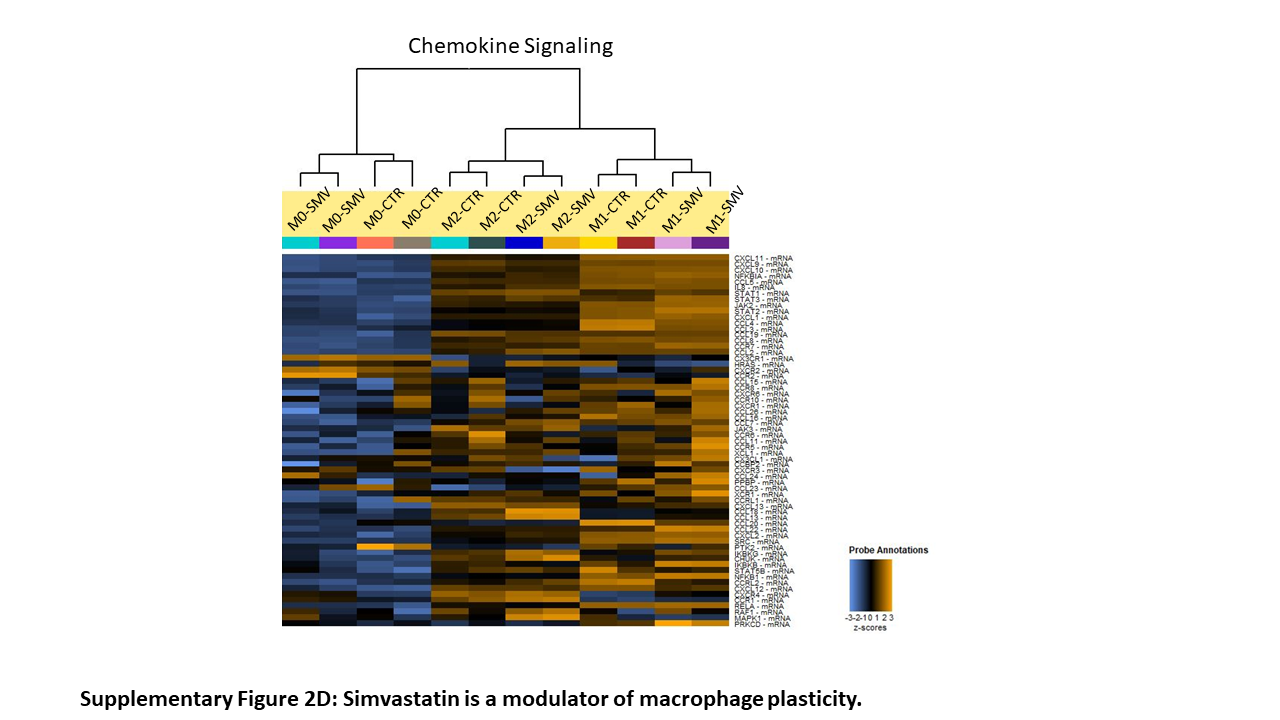

Supplement: Supplementary file 1 [file cells-12-01961-s001.zip › Supplementary Figure 2D.tif]
